# Supplementary material for: RodZ acts through MreBCD to activate the elongasome in Escherichia coli
Source: J Bacteriol. 2026 Aug 11;208(9):e00594-25. doi: 10.1128/jb.00594-25 (PMC13584716; doi:10.1128/jb.00594-25)
Supplement: Supplementary file 1 [file jb.00594-25-s0001.pdf]

Supplemental Information for

RodZ acts through MreBCD to activate the elongasome in *Escherichia coli*

Authors

Rui Zhan[1, 2], Han Gong[1, 2], Ying Li[1, 2], Yuanyuan Cui[1, 2], Xiangdong Chen[3], Joe Lutkenhaus[4], and Shishen Du[1, 2]*

Affiliation

1 State Key Laboratory of Metabolism and Regulation in Complex Organisms, College of Life Sciences, Wuhan University, Wuhan, Hubei, China

2 Hubei Key Laboratory of Cell Homeostasis, College of Life Sciences, Wuhan University, Wuhan, Hubei, China

3 State Key Laboratory of Virology and Biosafety, College of Life Sciences, Wuhan University, Wuhan, Hubei, China

4 Department of Microbiology, Molecular Genetics and Immunology, University of Kansas Medical Center, Kansas City, Kansas, USA

*** To whom correspondence should be addressed:**

Shishen Du

State Key Laboratory of Metabolism and Regulation in Complex Organisms, College of Life Sciences, Wuhan University, Wuhan, Hubei, China

e-mail: ssdu@whu.edu.cn

Supplementary Figure and Legends

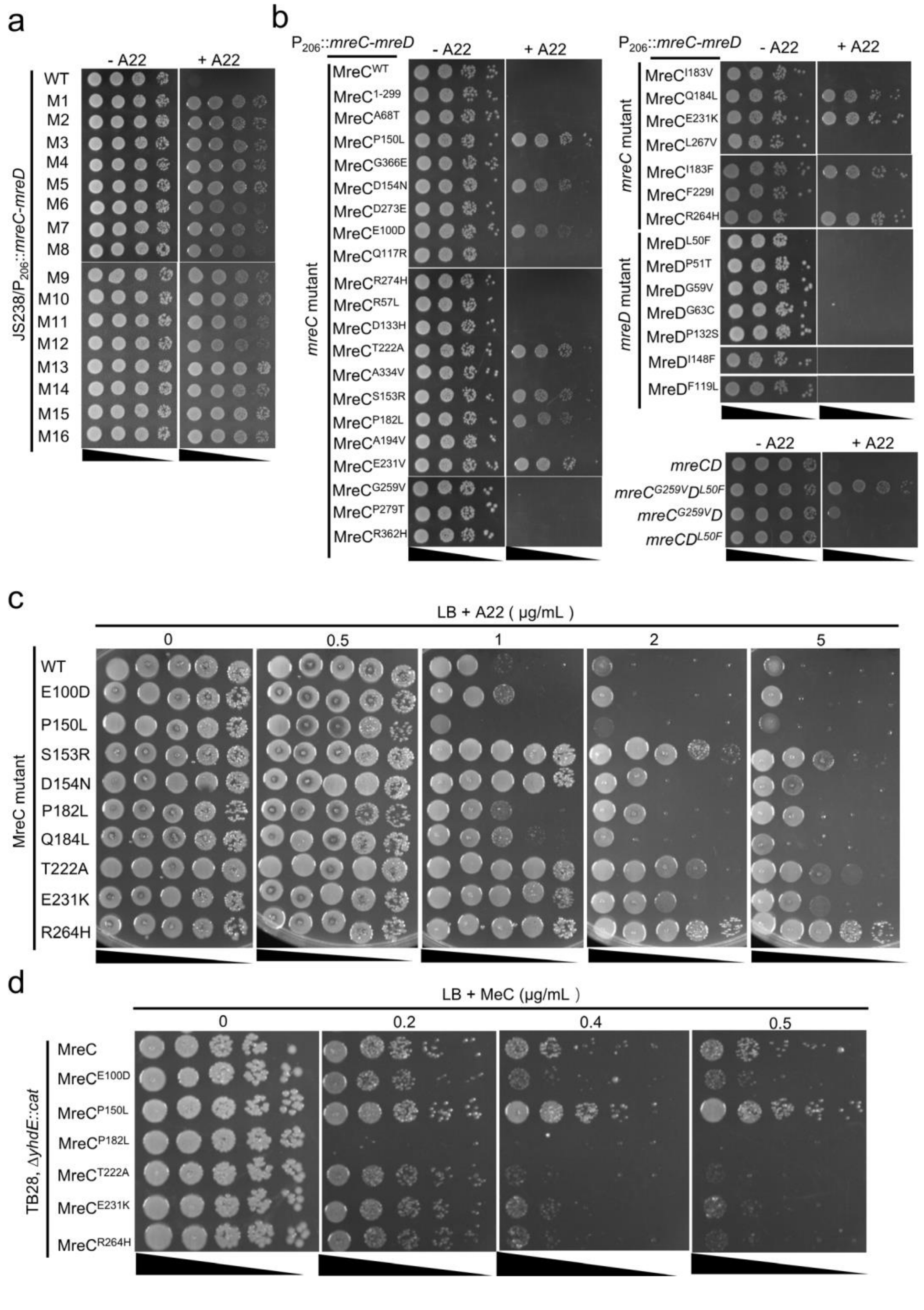

Supplementary Fig. 1 Screen for A22-resistant MreC mutants. (a) Transformants resistant to the MreB antagonist A22. The plasmid-based MreCD mutant library

pSD315^{M} (pDSW210, P_{206}::*mreCD*) was transformed into strain JS238 and transformants were selected on LB plates with or without A22 and 30 µM IPTG at 30°C. Plasmids from 16 transformants from the plates containing A22 were isolated and re-transformed into JS238. Transformants were tested on LB plates with or without A22 and 30 µM IPTG at 30°C to verify the A22 resistance was due to the transformed plasmids. (b) Determination of the substitutions providing resistance to A22. Plasmid pSD315 (pDSW210, P_{206}::*mreCD*) or its derivatives with different *mreC or mreD* alleles were transformed into strain JS238 and transformants were selected on LB plates with antibiotics at 37°C. Next day, the transformants were spotted onto the indicated media and incubated at 30°C overnight before photography. (c) MreC mutants provide resistance to A22. Strains (SD475 to SD504) containing the indicated *mreC* alleles at the native genomic locus were serially diluted and spotted on LB agar supplemented with different concentrations of A22. Plates were incubated at 30°C for 16 h prior to being photographed. (d) Most MreC mutants become more sensitive to PBP2 inhibitor mecillinam. The spot test was performed as in (c). The *mreC* alleles include *mreC*^{E100D}, *mreC*^{P150L}, *mreC*^{S153R}, *mreC*^{D154N}, *mreC*^{P182L}, *mreC*^{Q184L}, *mreC*^{T222A}, *mreC*^{E231K}, *mreC*^{R264H}.

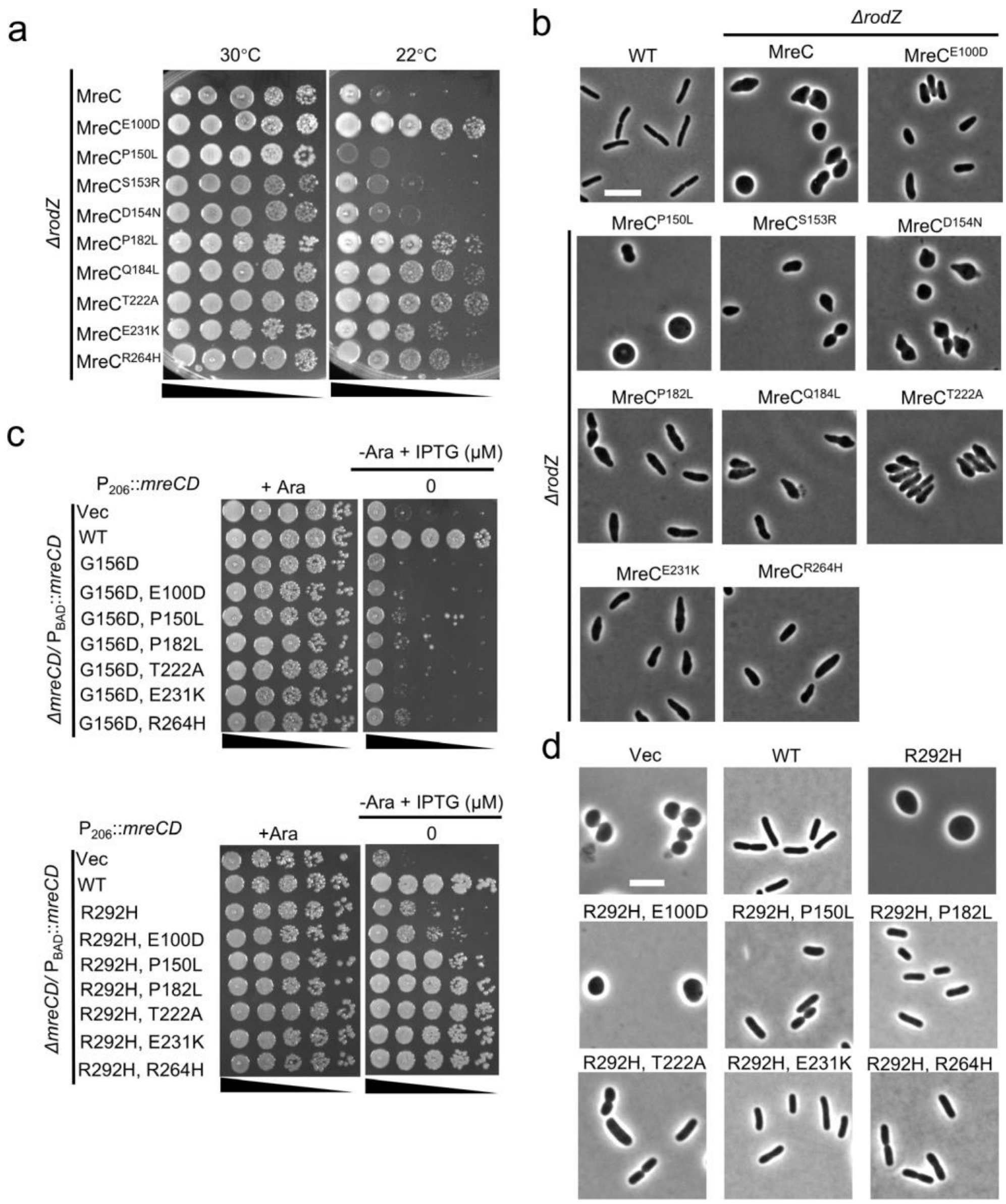

Supplementary Fig. 2 A22-resistant MreC mutations suppress the defects caused by deletion of *rodZ* or inactive *mreC* mutation. (a) MreC mutants rescued the growth of a *ΔrodZ* strain. Strains (SD541 to SD550) containing the indicated *mreC* alleles at the native genomic locus were serially diluted and spotted on LB agar supplemented with antibiotics. Plates were incubated at 22°C (non-permissive condition) for 40 h or 30°C (permissive condition) for 16 h prior to being photographed. The *mreC* alleles include *mreC*^{E100D}, *mreC*^{P150L}, *mreC*^{S153R}, *mreC*^{D154N}, *mreC*^{P182L}, *mreC*^{Q184L}, *mreC*^{T222A}, *mreC*^{E231K} and *mreC*^{R264H}. (b) MreC mutations partially suppress

the shape defect of *ΔrodZ* cells. Overnight cultures of the indicated strains (SD541 to SD550) were diluted 1:100 in 5 mL LB supplemented with kanamycin and grown to an OD_{600} of 0.4 to 0.6 at 30°C. Cells were immobilized on an agarose pad and imaged. Scale bar, 5 µm. (c) Activating MreC mutations are intragenic suppressors of $MreC^{R292H}$ but not $MreC^{G156D}$. (d) Activating MreC mutants suppress the cell shape defect caused by $MreC^{R292H}$. Overnight cultures of the strains from panel (c) were diluted 1:100 in fresh LB medium with antibiotics and 0.2% arabinose, grown at 37°C for 2-3 h. Cells were then collected by centrifugation and washed twice with fresh LB and resuspended in the same volume of LB medium. When the OD_{600} reached 0.4 to 0.6, these arabinose-free culture were then diluted 1:10 in fresh LB medium and IPTG was added to a final concentration of 60 µM, grown at 37°C about 3-4 hours. Cells were immobilized on 2% agarose pad for photography. Scale bar, 5 µm.

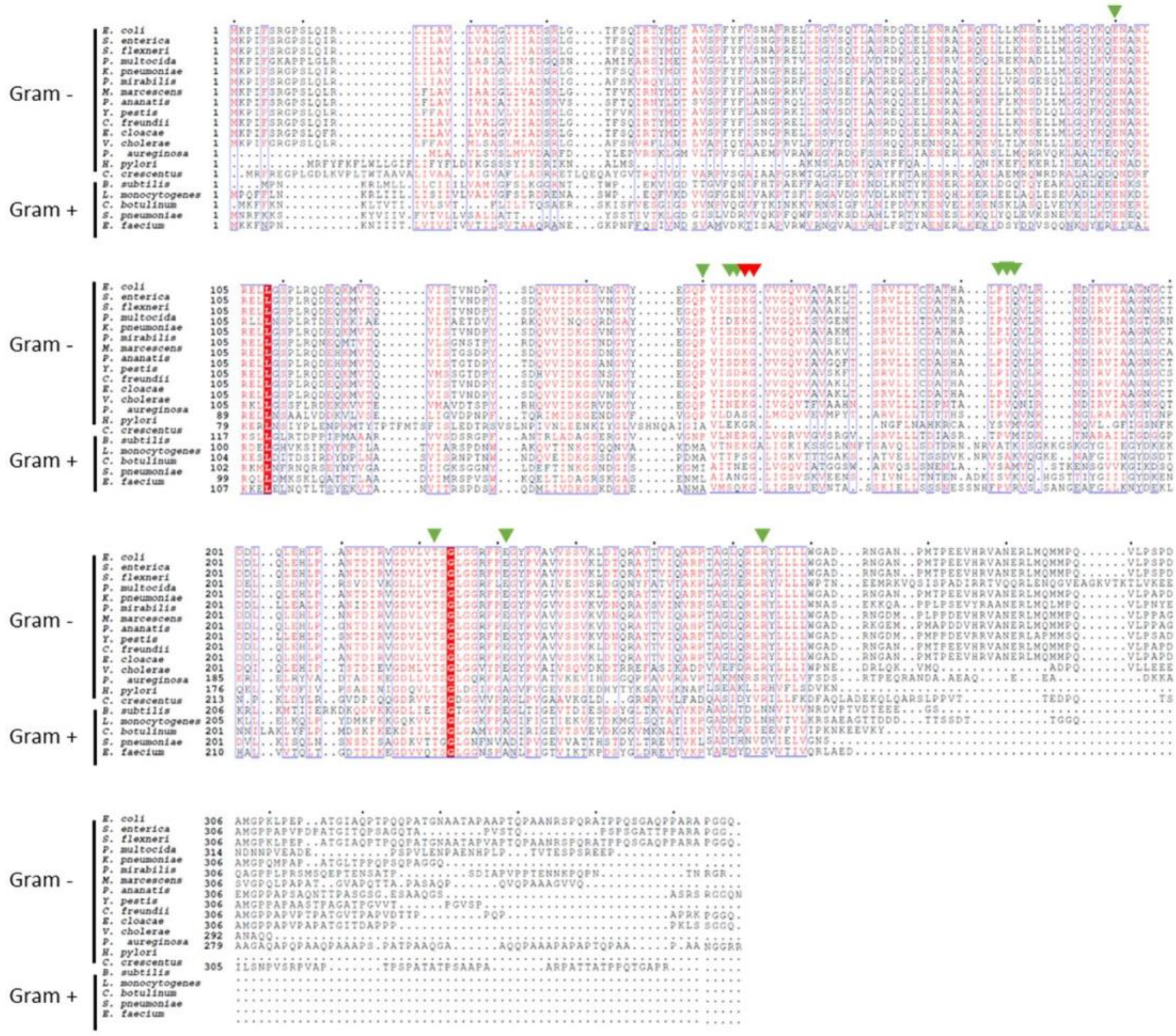

Supplementary Fig. 3 Alignment of the MreC proteins from diverse bacterial species. Amino acid sequences were obtained from Uniprot, aligned by Clustal Omega and then displayed using ESPRIPT: http://espript.ibcp.fr/. Green triangles indicated activating mutations, whereas red triangles indicated dominant-negative (inactive) mutations. MreC: *E. coli* (BAE77292.1), *S. enterica* (AMM01179.1), *S. flexneri* (KFZ99853.1), *P. multocida* (AIN48559.1), *K. pneumoniae* (CDO12223.1), *P. mirabilis* (KGA91538.1), *S. marcescens* (KFL03187.1), *P. ananatis* (CRH36157.1), *Y. pestis* (CAL22255.1), *C. freundii* (KFB98670.1), *E. cloacae* (KGB09506.1), *V. cholerae* (AKB03840.1), *P. aeruginosa* (NP_253170.1), *H. pylori* (CAX30066.1), *C. crescentus* (YP_002516986.1), *B. subtilis* (NP_390680.2), *L. monocytogenes* (NP_465072.1), *C. botulinum* (APH16565.1), *S. pneumoniae* (ARD37995.1), *E. faecium* (YP_006377167.1).

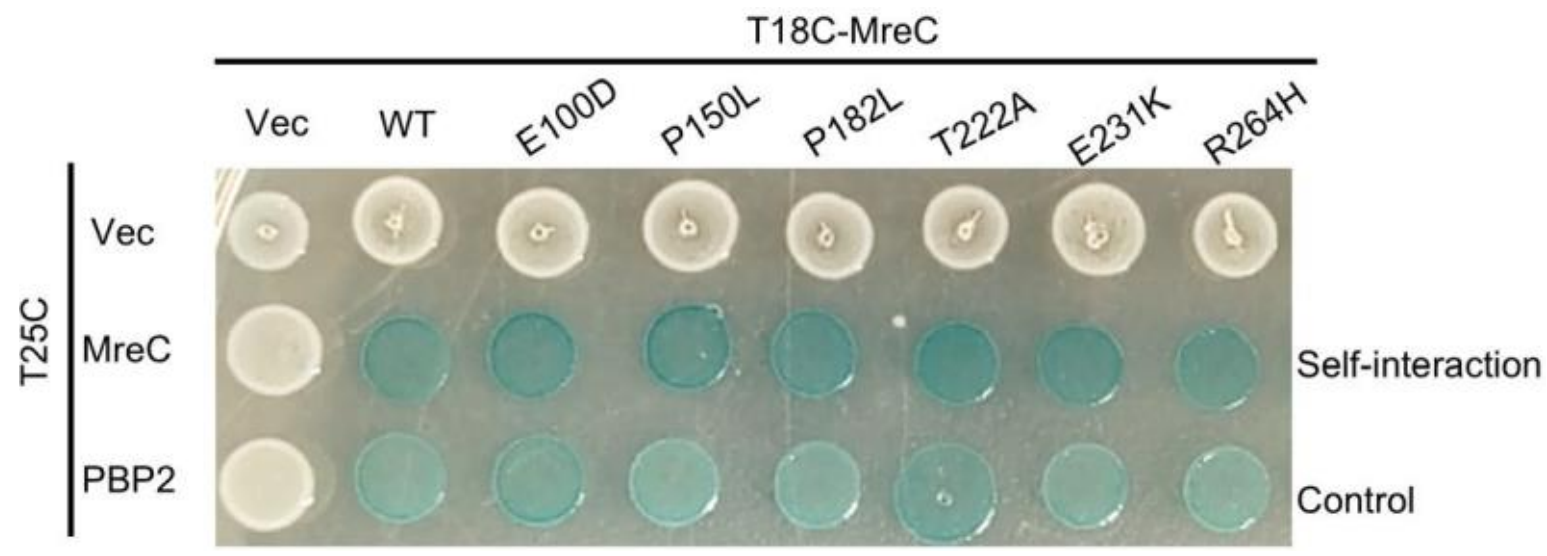

Supplementary Fig. 4 MreC mutants display normal self-interaction and interaction with PBP2 as indicated by BTH assay. Pairs of plasmids harboring T25- and T18- fusions of the indicated proteins or their variants were co-transformed into BTH101. The second row represented the detection of MreC self-interaction (mutant vs. mutant), while the third row represent the detection of MreC interaction with PBP2. The alleles of *mreC* include E100D, P150L, P182L, T222A, E231K and R264H. Note that both the T18C- and T25C- fusions of MreC contain the indicated mutation.

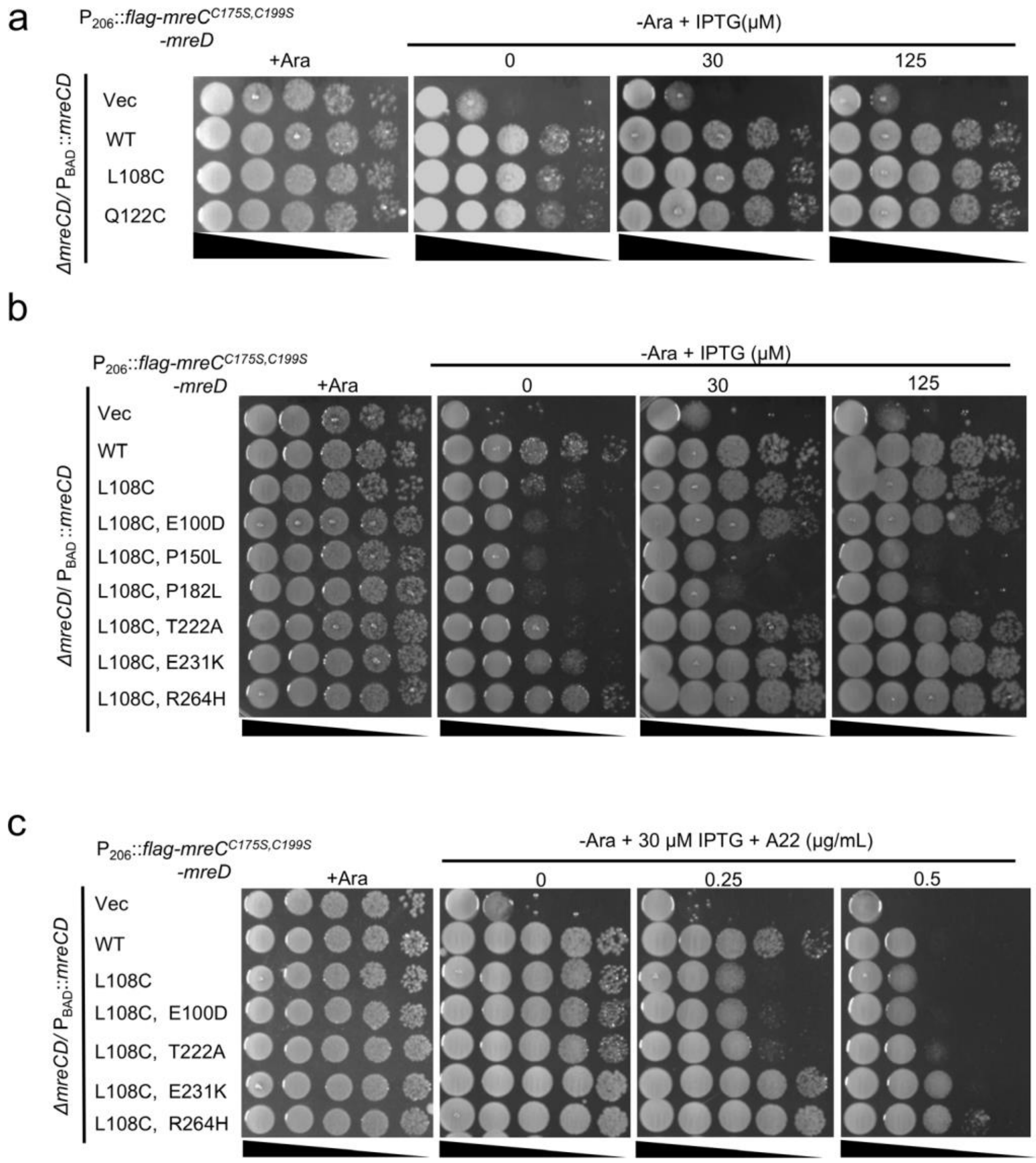

Supplementary Fig. 5 Complementation test and A22 resistance tests of MreC cysteine mutants. (a) Complementation test of MreC cysteine variants. (b) Complementation test of MreC mutants harboring the cysteine mutation and activating mutation. (c) A spot test of MreC cysteine variants' sensitivity to A22. Plates were incubated at 37°C overnight and photographed.

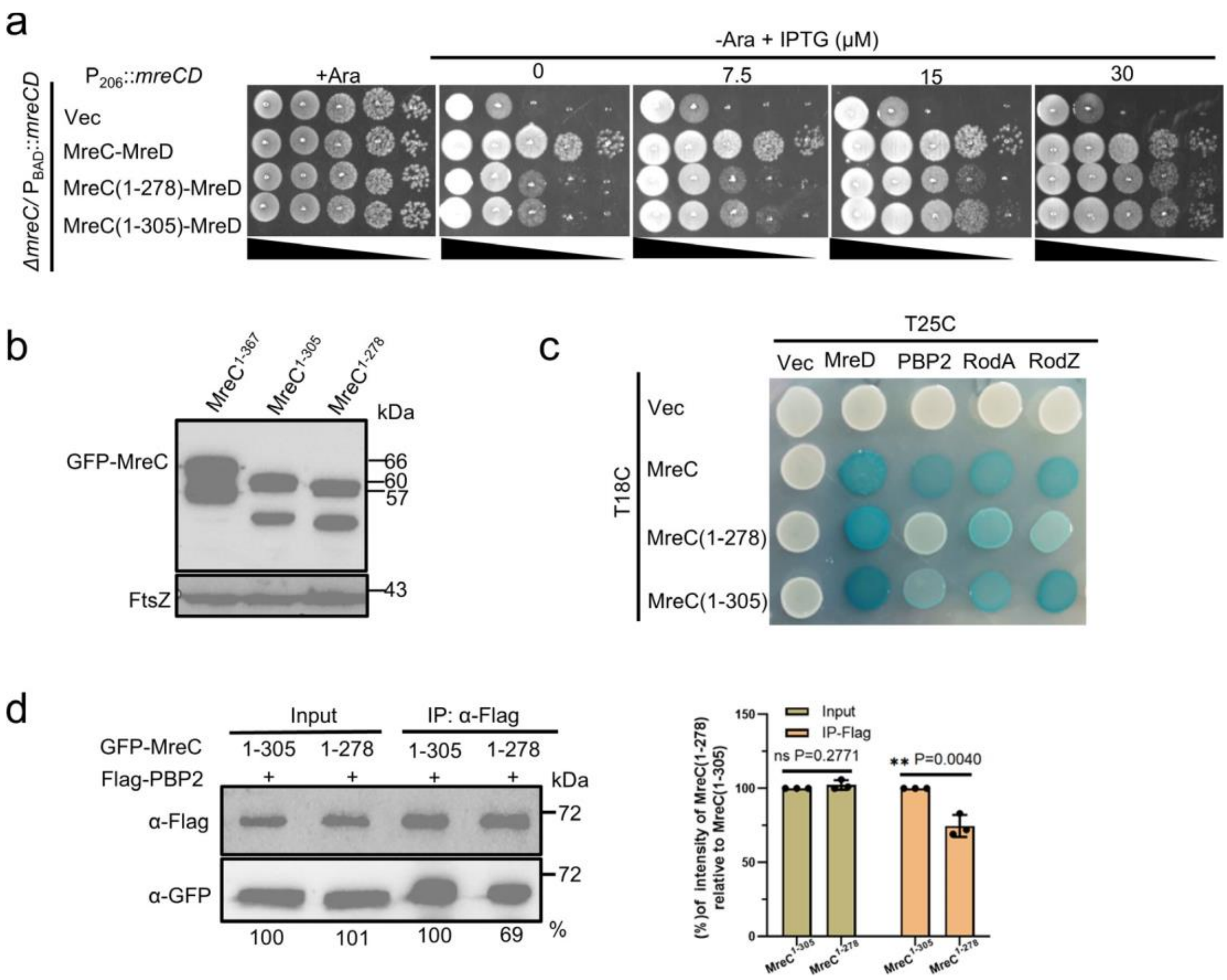

Supplementary Fig. 6 The deletion of the γ domain of MreC reduces the MreC-PBP2 interaction. (a) Complementation test of MreC truncated mutants. (b) Western blot to determine the stability of MreC mutant proteins. Detailed experimental procedure was provided in Materials and Methods. FtsZ was used as a loading control. Anti-GFP and Anti-FtsZ antibody was used at a dilution of 1/10,000. (c) Deletion of the γ domain of MreC impairs its interaction with PBP2 as indicated by BTH assay. Plasmid pairs were transformed into strain BTH101. The spot test was performed as in Fig. 3a. (d) MreC$^{1\text{-}278}$ displayed reduced interaction with PBP2 as indicated by Co-IP. Intensities of the bands were quantitated by Image J. Data are representative of three independent biological replicates. Statistical significance was determined using an unpaired t-test with Welch's correction. * $P < 0.1$, ** $P < 0.01$, *** $P < 0.001$; ns: non-significant.

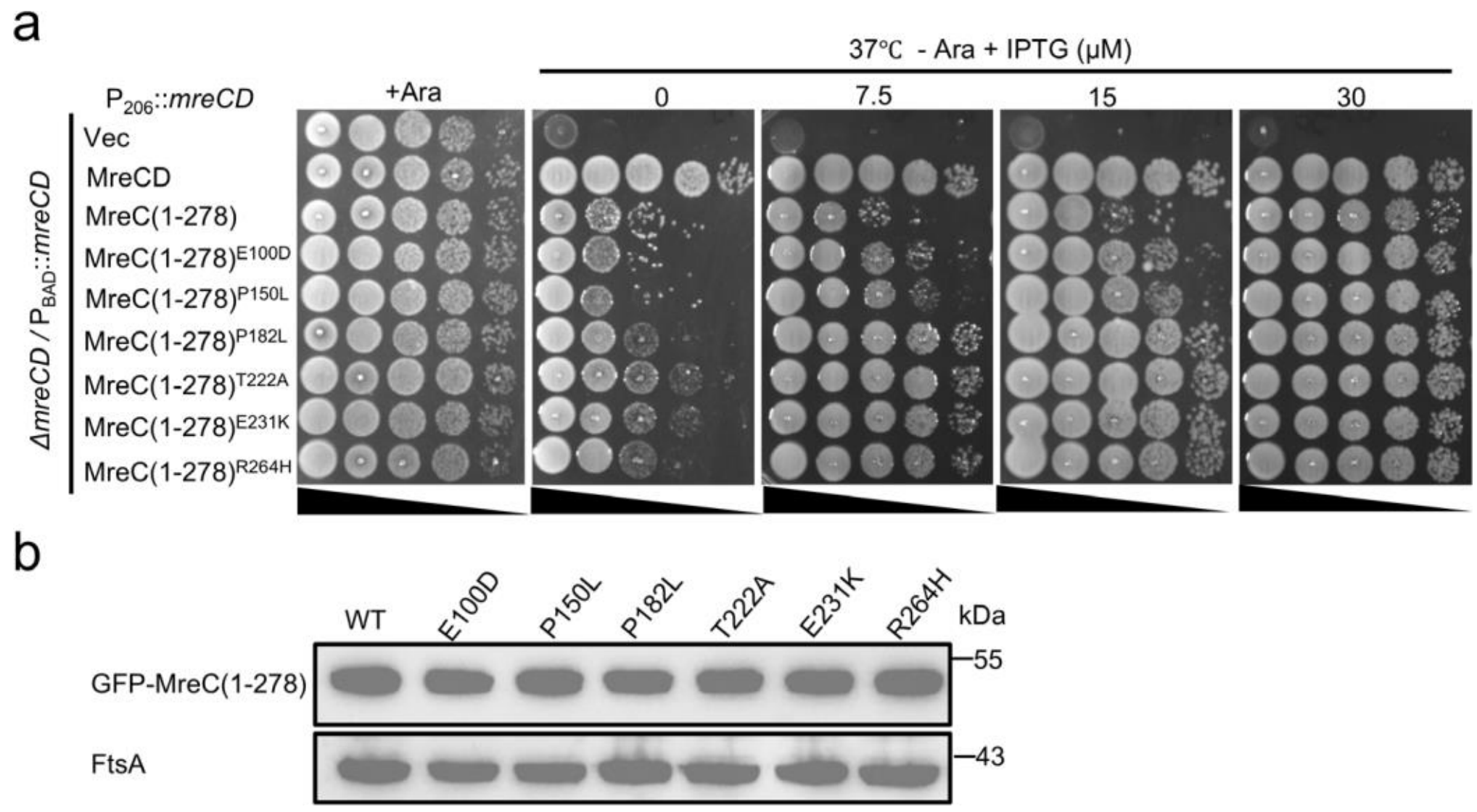

Supplementary Fig. 7 Activating *mreC* mutations suppress the growth defect caused by MreC$^{1\text{-}278}$. (a) Spot test of the rescuing effect of activating MreC mutations on MreC$^{1\text{-}278}$. (b) Western blotting assay to access the effect of activating mutations on the stability of MreC$^{1\text{-}278}$. Plasmids expressing *gfp-mreC$^{1\text{-}278}$* or its variants were transformed into TB28, samples were prepared for western blotting as in Fig. S6b. FtsA was used as a loading control.

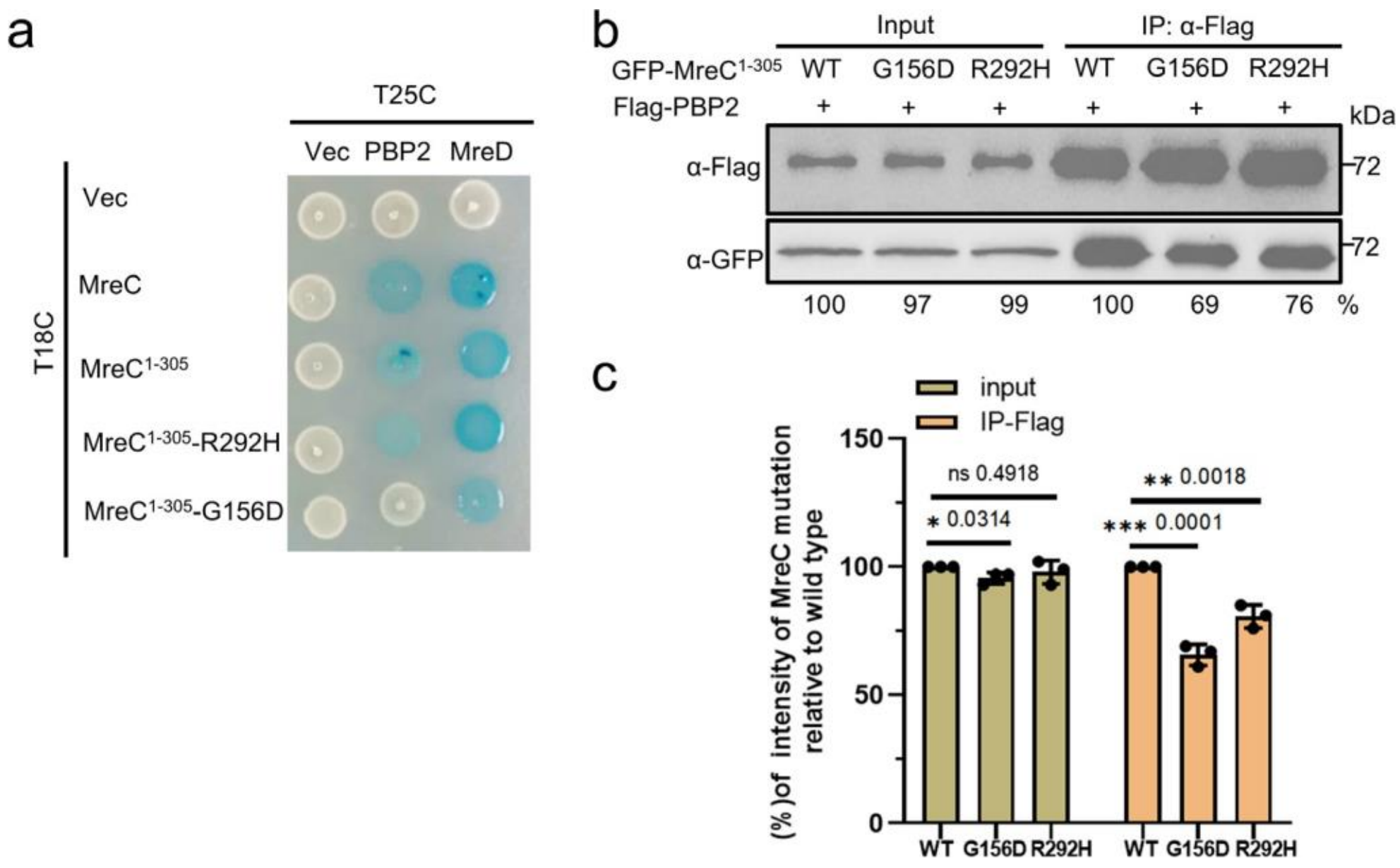

Supplementary Fig. 8 Inactivating MreC mutations impair the interaction between MreC and PBP2. (a) Inactivating MreC mutations reduced or eliminated the interaction between MreC$^{1\text{-}305}$ and PBP2 as indicated by BTH assay. Plasmid pairs expressing the T18 and T25 fusions were transformed into BTH101. The test was performed as in Fig. 3a. (b-c) Inactivating MreC mutations reduced the interaction between GFP-MreC$^{1\text{-}305}$ and Flag-PBP2 as indicated by Co-IP. A representative plot of three independent biological replicates was shown. Quantitation of the intensity of proteins was shown below the blot (c). The alleles of *mreC* include G156D and R292H. Statistical significance was determined using an unpaired t-test with Welch's correction. * $P < 0.1$, ** $P < 0.01$, *** $P < 0.001$; ns: non-significant.

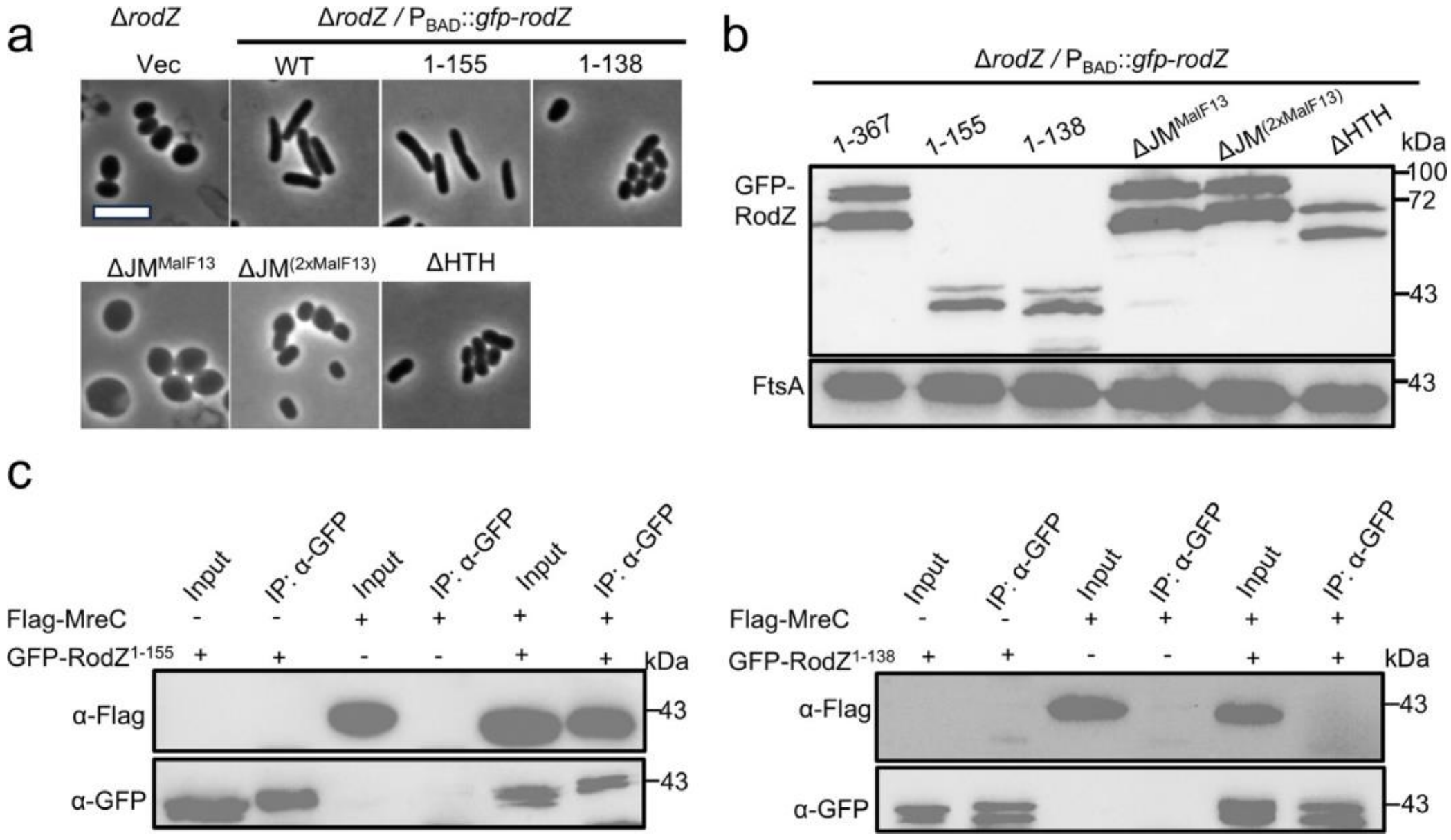

Supplementary Fig. 9 Characterization of RodZ variants. (a) Morphology of *ΔrodZ* strain expressing different RodZ variants. Overnight cultures of strain SD541 carrying plasmids pBAD18 (vec), pZR102 (RodZ), pZR103 ($RodZ^{1-155}$), pZR104 ($RodZ^{1-138}$), pZR105 ($RodZΔJM^{MalF13}$), pZR106 ($RodZΔJM^{2×MalF13}$) and pZR107 (RodZΔHTH) were grown in LB, diluted 1:100 into the same medium supplemented with 0.2% arabinose, and grown at 30°C until OD_{600} reached about 0.4-0.6. Then the cultures were diluted 1:20 in the same medium supplemented with 0.05% arabinose again, and grown at 30°C until OD_{600} =0.4-0.6. Cells were collected, fixed and then imaged. Scale bar, 5 µm. (b) Western blot to determine the protein level of GFP-RodZ mutants. Details about the western blot is described in Materials and Methods. Experiments were repeated three times and a representative blot image was shown. (c) $RodZ^{1-155}$ but not $RodZ^{1-138}$ interacts with MreC *in vivo* as indicated by Co-IP experiments. The experiments were performed as in Fig. 5d, except for the antibodies used. Supernatants were incubated with rabbit GFP antibodies coated magnetic beads, while the following western blotting analysis used mouse GFP antibody and Flag antibody.

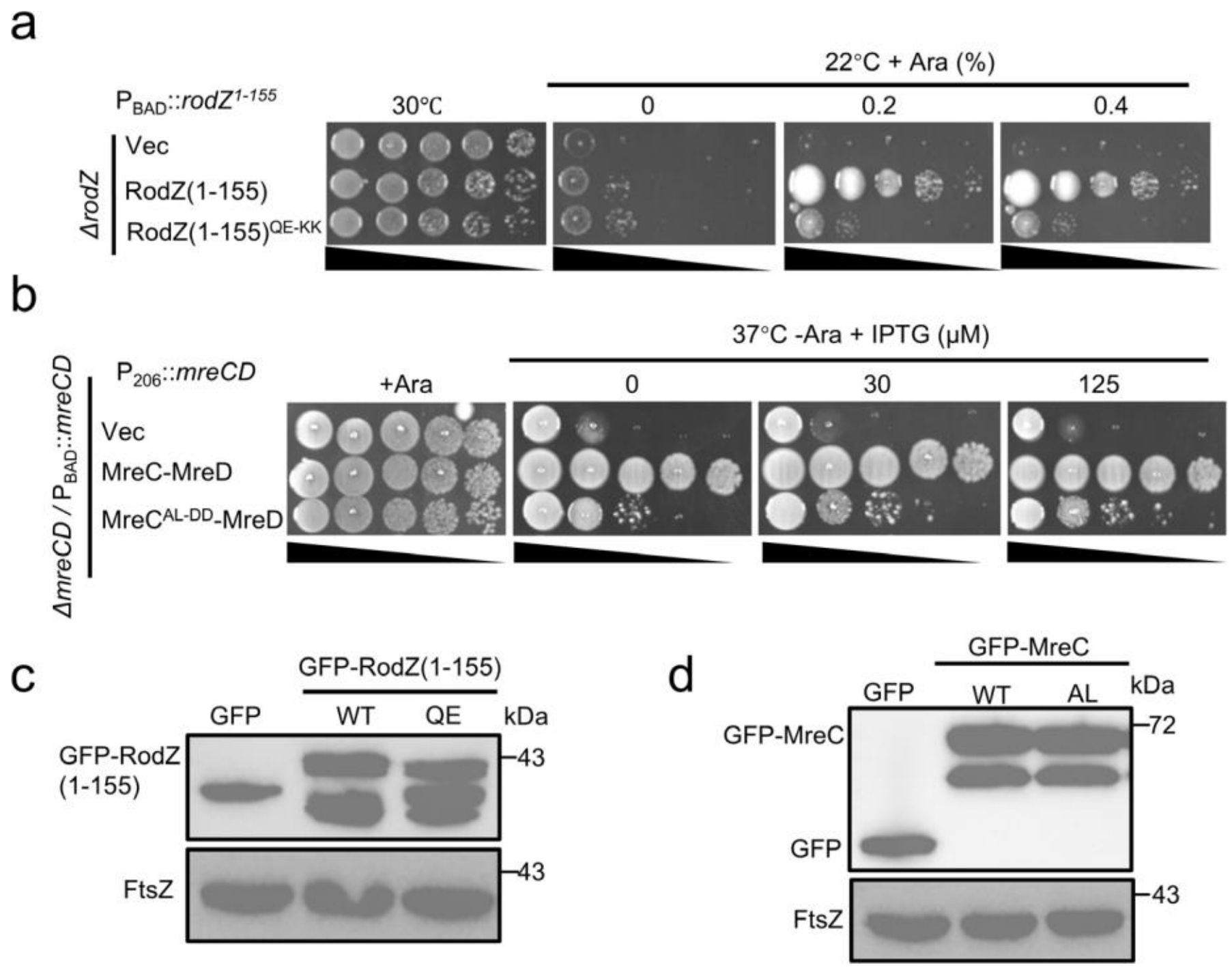

Supplementary Fig. 10 Disruption of the interaction between RodZ and MreC affects elongasome function. (a) Introduction of mutations into RodZ to disrupt its interaction with MreC$^{1\text{-}155}$ resulted in growth defects. The indicated strains were grown and prepared for spot tests as described in the legend for Fig. 6b. (b) Introduction of mutations into MreC to disrupt its interaction with RodZ resulted in growth defects. Plasmid pDSW210, pSD315 or its derivatives carrying *mreC* alleles were transformed into strain SD464 on LB plates with ampicillin, kanamycin, chloramphenicol and 0.2% arabinose. The spot test was performed as in Fig. S5a. (c-d) Western blot to determine the protein level of GFP-RodZ mutants (c) and GFP-MreC mutants (d). Details about the western blot was described in Methods. Experiments were repeated three times and a representative blot was shown.

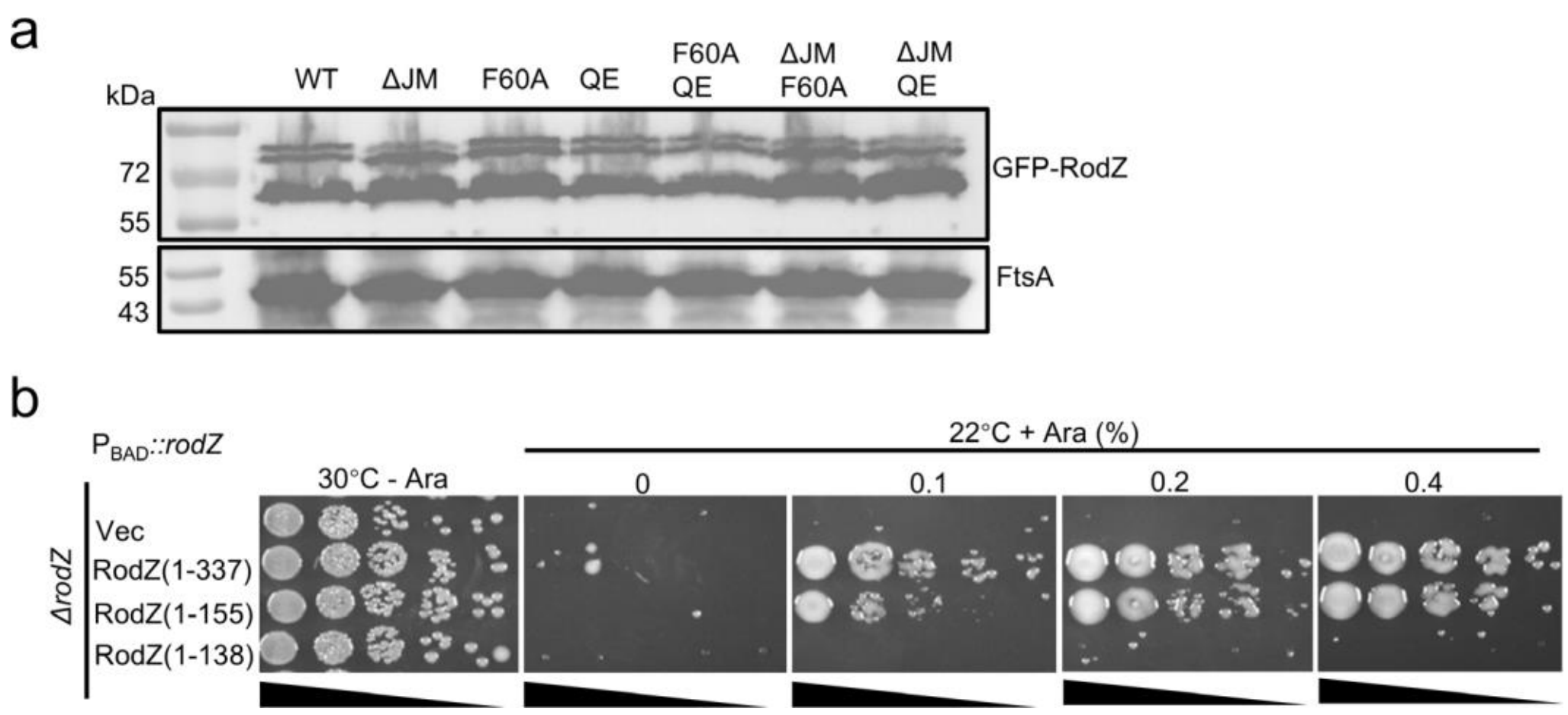

Supplementary Fig. 11 Functional characterization of RodZ variants. (a) Western blot to determine the protein level of RodZ variants. Details about the western blot was described in Methods. Experiments were repeated three times and a representative blot was shown. (b) RodZ1-138 failed to complement a *ΔrodZ* strain. Plasmid pBAD18, pZR95 (P_{BAD}::*rodZ*), pZR96 (P_{BAD}::*rodZ*$^{1-155}$) and pZR97 (P_{BAD}::*rodZ*$^{1-138}$) were transformed into strain SD541 on LB plates at 30˚C. The next day, single transformants were subjected to spot tests. Plates were incubated at 22°C for 40 hours or at 30˚C overnight and photographed

Supplementary Text

Strain Construction

SD464

Strain SD464 (TB28, *mreCD<>aph* / pSD352 (P_{BAD}::*mreCD*)) was constructed by P1 transduction(1) of the *mreCD*<>aph cassette from P2468 ([PB103, *mreCD<>aph* / *ColE1, tet sidA*]) into strain TB28/ pSD352. Transductants were selected on LB plates with 25 µg/mL kanamycin, 15 µg/mL chloramphenicol, 8 mM sodium citrate and 0.2% arabinose at 37°C. Deletion of *mreCD* was verified by arabinose-dependent growth and loss of rod-shape upon the removal of arabinose.

SD475

Strain SD475 (TB28, *yhdE<>cat*) was constructed by P1 transduction of the *yhdE::cat cassette* from DS452 (BW25113, *yhdE*::*cat*) into strain TB28. Transductants were selected on LB plates with 15 µg/mL chloramphenicol and 8 mM sodium citrate at 37°C.

SD495 to SD504

Strains SD495 to SD504 were obtained by replacing the chromosomal $mreC^{WT}$ allele of SD475 with respective *mreC* alleles from plasmid pZR4 ($pSC101^{ts}$, *mreC**)(2). Presence of the desired allele in each strain was verified by amplification and sequencing of the chromosomal DNA using primers 5-MreC-SeqF and 3-MreD-seqR.

SD541-SD550

Strains carrying the *rodZ<>aph* cassette were obtained by P1-mediated transduction of *rodZ<>aph* cassette from P5439 ([TB28, *rodZ<>aph* / *ColE1, bla* $lacI^q$ P_{lac}::*rodZ*]) into respective strains (Table S1). Transductants were selected on LB plates containing 25 µg/mL kanamycin, 15 µg/mL chloramphenicol and 8 mM sodium citrate at 37°C. Deletion of *rodZ* was verified by the loss of rod-shape of cells.

RZ7

Strain RZ7 was constructed by integrating the plasmid pHC897 (*attλ cat* $lacI^q$ P_{lac}::*mreB'-mNeonGreen-'mreB*) into strain TB28 / pINT-ts via electroporation(3). Cells

carrying the CRIM helper plasmid (pINT-ts) were grown in 5 mL of LB cultures with ampicillin at 30°C to OD_{600} reached about 0.4 to 0.6 and then made electrocompetent. Following electroporation, cells were suspended in LB without ampicillin, incubated at 37°C for 1 h to cure the temperature-sensitive plasmid, and then spread onto LB plate with 15 μg/mL chloramphenicol and incubated at 37°C. Next day, colonies were purified once non-selectively and then tested for antibiotic resistance for stable integration and loss of the helper plasmid and by PCR for copy number.

RZ8 to RZ14

Strain RZ8 to RZ14 was constructed by transforming with pCP20 to eliminate the antibiotic resistance gene *aph* of strains SD495 to SD504. Ampicillin-resistant transformants were selected at 30°C, purified once at 42°C and then tested for the loss of kanamycin resistance and loss of pCP20.

RZ15 to RZ21

Strains carrying the P_{lac}::^{SW}*mreB-mNeonGreen* cassette were obtained by P1-mediated transduction of P_{lac}::^{SW}*mreB-mNeonGreen* cassette from RZ7 (TB28, *attλ cat lacI*^{q} P_{tac}*::* ^{SW}*mreB-mNeonGreen*) into respective strains (RZ8 to RZ14). Transductants were selected on LB plates containing 15 μg/mL chloramphenicol and 8 mM sodium citrate at 37°C. Colonies were purified once and then tested for chloramphenicol resistance and green fluorescence.

Plasmids Construction

The plasmid pSD315 (pDSW210, *bla* P_{206}::*mreCD*) was constructed by ligation of an EcoRI/HindIII digested DNA fragment containing *mreC-mreD* into pDSW210 digested with the same enzymes. The DNA fragment was amplified from W3110 chromosomal DNA using primers 5-EcoRI-MreC and 3-mreD-HindIII. Derivatives of pSD315 carrying different alleles of *mreC* were obtained by site-directed mutagenesis using primer pairs listed in Supplementary Table S4.

The plasmid pSD315-1-278 (pDSW210, *bla* P_{206}::*mreC*$^{1-278}$-*mreD*) was constructed by ligation of an EcoRI/HindIII digested DNA fragment containing *mreC*$^{1-278}$-*mreD* into pDSW210 digested with the same enzymes. The *mreC*$^{1-278}$-*mreD* DNA fragment was obtained by overlap extension PCR. Two DNA fragments containing *mreC*$^{1-278}$ and *mreD* were amplified from pSD315 using primer pairs 5-EcoRI-MreC/3-mreD-mreC-278 and 5-MreC-278-MreD/3-mreD-HindIII. Derivatives of pSD315-1-278 carrying different alleles of *mreC*$^{1-278}$ were obtained by site-directed mutagenesis using primer pairs listed in Supplementary Table S4.

The plasmid pSD315-1-305 (pDSW210, *bla* P_{206}::*mreC*$^{1-305}$-*mreD*) was constructed by ligation of an EcoRI/HindIII digested DNA fragment containing *mreC*$^{1-305}$-*mreD* into pDSW210 digested with the same enzymes. The *mreC*$^{1-305}$-*mreD* DNA fragment was obtained by overlap extension PCR. Two DNA fragments containing *mreC*$^{1-305}$ and *mreD* were amplified from pSD315 using primer pairs 5-EcoRI-MreC/3-mreD-mreC-305 and 5-MreC-305-MreD/3-mreD-HindIII.

The plasmid pSD352 (pBAD33, p15A P_{BAD}::*mreC-mreD*, *Cam*^{R}) was constructed by ligation of an XbaI/HindIII digested DNA fragment containing *mreC-mreD* into pBAD33 digested with the same enzymes. The DNA fragment was amplified from W3110 chromosomal DNA using primers 5-XbaI-mreC and HindIII-mreD-3.

The plasmid pZR4 (pSC101^{ts}, *mreC-mreD, Spc*^{R}) was constructed by ligation of an EcoRI/HindIII digested DNA fragment containing *mreC-mreD* into pHGB2 digested with the same enzymes. The DNA fragment was amplified from plasmid pSD315 using primers 5-HindIII-MreC and 3-EcoRI-MreD. Derivatives of pZR4 carrying different alleles of *mreC* were obtained by site-directed mutagenesis using primer pairs listed in Supplementary Table S4.

The plasmid pZR7 (pMB1, *bla* P_{lac}::*t18-mreC*) was constructed by ligation of an KpnI/XbaI digested DNA fragment containing *mreC* into pUT18C digested with the same enzymes. The DNA fragment was amplified from plasmid pSD315 using primers T18C-XbaI-mreC and mreC-KpnI-T18C. Derivatives of pZR7 carrying different alleles

of *mreC* were obtained by site-directed mutagenesis using primer pairs listed in Supplementary Table S4.

The plasmid pZR7-1-305 (pMB1, *bla* P_{lac}::*t18-mreC*$^{1\text{-}305}$) was constructed by ligation of an XbaI/KpnI digested DNA fragment containing *mreC*$^{1\text{-}305}$ into pUT18C digested with the same enzymes. The DNA fragment was amplified from plasmid pSD315 using primers T18C-XbaI-mreC and 3-MreC-1-305-KpnI. Derivatives of pZR7-1-305 carrying different alleles of *mreC*$^{1\text{-}305}$ were obtained by site-directed mutagenesis using primer pairs listed in Supplementary Table S4.

The plasmid pZR7-1-278 (pMB1, *bla* P_{lac}::*t18-mreC*$^{1\text{-}278}$) was constructed by ligation of an XbaI/KpnI digested DNA fragment containing *mreC*$^{1\text{-}278}$ into pUT18C digested with the same enzymes. The DNA fragment was amplified from plasmid pSD315 using primers T18C-XbaI-mreC and 3-MreC-1-278-KpnI. Derivatives of pZR7-1-278 carrying different alleles of *mreC*$^{1\text{-}278}$ were obtained by site-directed mutagenesis using primer pairs listed in Supplementary Table S4.

The plasmid pZR8 (pACYC184, *kan* P_{lac}::*t25-mreC*) was constructed by ligation of an KpnI/XbaI digested DNA fragment containing *mreC* into pKT25 digested with the same enzymes. The DNA fragment was amplified from plasmid pSD315 using primers T18C-XbaI-mreC and mreC-KpnI-T18C.

The plasmid pZR10 (pACYC184, *kan* P_{lac}::*t25-mreD*) was constructed by ligation of an XbaI/kpnI digested DNA fragment containing *mreD* into pKT25 digested with the same enzymes. The DNA fragment was amplified from plasmid pSD315 using primers T25C-XbaI-mreD and mreD-KpnI-T25C.

The plasmid pZR12 (pACYC184, *kan* P_{lac}::*t25-pbp2*) was constructed by ligation of an XbaI/EcoRI digested DNA fragment containing *pbp2* into pKT25 digested with the same enzymes. The DNA fragment was amplified from W3110 chromosomal DNA using primers T25-XbaI-PBP2 and PBP2-EcoRI-T25C.

The plasmid pZR17 (pMB1, *bla* P_{lac}::*t18-rodZ)* was constructed by ligation of an XbaI/KpnI digested DNA fragment containing *rodZ* into pUT18C digested with the

same enzymes. The DNA fragment was amplified from W3110 chromosomal DNA using primers T18C-XbaI-RodZ and RodZ-KpnI-T18C.

The plasmid pZR17-1-155 (pMB1, *bla* P_{lac}::*t18-rodZ*$^{1-155}$) was constructed by ligation of an XbaI/KpnI digested DNA fragment containing *rodZ*$^{1-155}$ into pUT18C digested with the same enzymes. The DNA fragment was amplified from W3110 chromosomal DNA using primers T18C-XbaI-RodZ and 3-RodZ155-kpnI. Derivatives of pZR17-1-155 carrying different alleles of *rodZ*$^{1-155}$ were obtained by site-directed mutagenesis using primer pairs listed in Table S4.

The plasmid pZR18 (pACYC184, *kan* P_{lac}::*t25-rodZ*) was constructed by ligation of an XbaI/KpnI digested DNA fragment containing *rodZ* into pKT25 digested with the same enzymes. The DNA fragment was amplified from W3110 chromosomal DNA using primers T25C-XbaI-RodZ and RodZ-KpnI-T25C.

The plasmid pZR18-1-155 (pACYC184, *kan* P_{lac}::*t25-rodZ*$^{1-155}$) was constructed by ligation of an XbaI/KpnI digested DNA fragment containing *rodZ*$^{1-155}$ into pKT25 digested with the same enzymes. The DNA fragment was amplified from W3110 chromosomal DNA using primers T25C-XbaI-RodZ and RodZ-1-155-KpnI-T25C.

The plasmid pZR26 (ColE1, P_{lac}::*mreB'-t25-mreB', Amp*^{R}) was constructed by ligation of an XhoI/AscI digested DNA fragment containing *t25c* fragment into pHC892 digested with the same enzymes. The DNA fragment was amplified from pKT25 using primers 5-XhoI-t25 and 3-T25-AscI.

The plasmid pZR28 (pACYC184, *kan* P_{lac}::*mreB'-t25c-mreB')* was constructed by ligation of an XbaI/ClaI digested DNA fragment containing *t25-mreB'-t25c* fragment into pKNT25 digested with the same enzymes. The DNA fragment was amplified from pZR26 using primers pUT18-XbaI-mreB-U and pUT18-ClaI-MreB-U.

The plasmid pZR82 (pDSW209, *bla* P_{206}::*gfp-rodZ*) was constructed by ligation of an KpnI/XbaI digested DNA fragment containing *rodZ* into pDSW209 digested with the same enzymes. The DNA fragment was amplified from W3110 chromosomal DNA using primers 5-kpnI-GFP-RodZ and 3-RodZ-XbaI.

The plasmid pZR83 (pDSW209, *bla* P_{206}::*gfp-rodZ*$^{1\text{-}138}$) was constructed by ligation of an KpnI/XbaI digested DNA fragment containing *rodZ*$^{1\text{-}138}$ into pDSW209 digested with the same enzymes. The DNA fragment was amplified from W3110 chromosomal DNA using primers 5-kpnI-GFP-RodZ and 3-RodZ-1-138-XbaI. Derivatives of pZR83 carrying different alleles of *rodZ*$^{1\text{-}138}$ were obtained by site-directed mutagenesis using primer pairs listed in Supplementary Table S4.

The plasmid pZR84 (pDSW209, *bla* P_{206}::*gfp-rodZ*$^{1\text{-}155}$) was constructed by ligation of an KpnI/XbaI digested DNA fragment containing *rodZ*$^{1\text{-}155}$ into pDSW209 digested with the same enzymes. The DNA fragment was amplified from W3110 chromosomal DNA using primers 5-kpnI-GFP-RodZ and 3-RodZ-1-155-XbaI. Derivatives of pZR84 carrying different alleles of *rodZ*$^{1\text{-}155}$ were obtained by site-directed mutagenesis using primer pairs listed in Supplementary Table S4.

The plasmid pZR85 (pDSW209, *bla* P_{206}::*gfp-rodZ*$^{\Delta HTH(1\text{-}82)}$) was constructed by ligation of an KpnI/XbaI digested DNA fragment containing *rodZ*$^{\Delta HTH(1\text{-}82)}$ into pDSW209 digested with the same enzymes. The DNA fragment was amplified from W3110 chromosomal DNA using primers 5-kpnI-RodZΔHTH-GFP and 3-RodZ-XbaI. Derivatives of pZR85 carrying different alleles of *rodZ*$^{\Delta HTH}$ were obtained by site-directed mutagenesis using primer pairs listed in Supplementary Table S4.

The plasmid pZR86 (pDSW209, *bla* P_{206}::*gfp-rodZ(1-155)ΔJM*^{MalF13}) was constructed by ligation of an KpnI/XbaI digested DNA fragment containing *rodZ(1-155)ΔJM*^{MalF13} into pDSW209 digested with the same enzymes. The *rodZ(1-155)ΔJM*^{MalF13} DNA fragment was obtained by overlap extension PCR. Two DNA fragments were amplified from pZR84 using primer pairs 5-GFP-RodZ1-155-U-KpnI/ 3-MalF13-RodZ and 5-MalF13-RodZ/ 3-XbaI-RodZ-1-155-U. The amino-acid sequence of the MalF(1-14): ATGGATGTCATTAAAAAGAAACATTGGTGGCAAAGCGACGCG.

The plasmid pZR87 (pDSW209, *bla* P_{206}::*gfp-rodZ(1-155)ΔJM*$^{(2xMalF13)}$) was constructed by ligation of an KpnI/XbaI digested DNA fragment containing *rodZ(1-155)ΔJM*$^{(2xMalF13)}$ into pDSW209 digested with the same enzymes. The *rodZ(1-*

155)ΔJM$^{(2xMalF13)}$ DNA fragment was obtained by overlap extension PCR. Two DNA fragments were amplified from pZR86 using primer pairs 5-GFP-RodZ1-155-U-KpnI/ MalF13-DOWN-UP and MalF13-UP-DOWN/ 3-XbaI-RodZ-1-155-U.

The plasmid pZR88 (pDSW209, *bla* P_{206}::*gfp-rodZΔJM^{MalF13}*) was constructed by ligation of an KpnI/XbaI digested DNA fragment containing *rodZΔJM^{MalF13}* into pDSW209 digested with the same enzymes. The *rodZΔJM^{MalF13}* DNA fragment was obtained by overlap extension PCR. Two DNA fragments were amplified from pZR82 using primer pairs 5-GFP-RodZ-U-KpnI/ 3-MalF13-RodZ and 5-MalF13-RodZ/ 3-XbaI-RodZ-U.

The plasmid pZR89 (pDSW209, *bla* P_{206}::*gfp-rodZΔJM$^{(2xMalF13)}$*) was constructed by ligation of an KpnI/XbaI digested DNA fragment containing *rodZΔJM$^{(2xMalF13)}$* into pDSW209 digested with the same enzymes. The *rodZΔJM$^{(2xMalF13)}$* DNA fragment was obtained by overlap extension PCR. Two DNA fragments were amplified from pZR88 using primer pairs 5-GFP-RodZ-U-KpnI/ MalF14-DOWN-UP and MalF14-UP-DOWN/ 3-XbaI-RodZ-U.

The plasmid pZR95 (pBAD18, P_{BAD}::*rodZ*, *Amp^{R}*) was constructed by ligation of a KpnI/XbaI digested DNA fragment containing *rodZ* into pBAD18 digested with the same enzymes. The DNA fragment was amplified from W3110 chromosomal DNA using primers 5-KpnI-RodZ and 3-RodZ-XbaI.

The plasmid pZR96 (pBAD18, P_{BAD}::*rodZ$^{1-155}$*, *Amp^{R}*) was constructed by ligation of an KpnI/XbaI digested DNA fragment containing *rodZ$^{1-155}$* into pBAD18 digested with the same enzymes. The DNA fragment was amplified from W3110 chromosomal DNA using primers 5-KpnI-RodZ and 3-RodZ-1-155-XbaI. Derivatives of pZR96 carrying different alleles of *rodZ$^{1-155}$* were obtained by site-directed mutagenesis using primer pairs listed in Supplementary Table S4.

The plasmid pZR97 (pBAD18, P_{BAD}::*rodZ$^{1-138}$*, *Amp^{R}*) was constructed by ligation of an KpnI/XbaI digested DNA fragment containing *rodZ$^{1-138}$* into pBAD18 digested with the

same enzymes. The DNA fragment was amplified from W3110 chromosomal DNA using primers 5-KpnI-RodZ and 3-RodZ-1-138-XbaI.

The plasmid pZR102 (pBAD18, P_{BAD}::*gfp-rodZ*, *Amp^R*) was constructed by ligation of an KpnI/XbaI digested DNA fragment containing *gfp-rodZ* into pBAD18 digested with the same enzymes. The DNA fragment was amplified from pZR82 chromosomal DNA using primers 5-KpnI-GFP-U-pBAD18 and 3-XbaI-RodZ-U-pBAD18. Derivatives of pZR102 carrying different alleles of *gfp-rodZ* were obtained by site-directed mutagenesis using primer pairs listed in Supplementary Table S4.

The plasmid pZR103 (pBAD18, P_{BAD}::*gfp-rodZ$^{1\text{-}155}$*, *Amp^R*) was constructed by ligation of an KpnI/XbaI digested DNA fragment containing *gfp-rodZ$^{1\text{-}155}$* into pBAD18 digested with the same enzymes. The DNA fragment was amplified from pZR84 chromosomal DNA using primers 5-KpnI-GFP-U-pBAD18 and 3-XbaI-RodZ1-155-U-pBAD18. Derivatives of pZR103 carrying different alleles of *gfp-rodZ$^{1\text{-}155}$* were obtained by site-directed mutagenesis using primer pairs listed in Supplementary Table S4.

The plasmid pZR104 (pBAD18, P_{BAD}::*gfp-rodZ$^{1\text{-}138}$*, *Amp^R*) was constructed by ligation of an KpnI/XbaI digested DNA fragment containing *gfp-rodZ$^{1\text{-}138}$* into pBAD18 digested with the same enzymes. The DNA fragment was amplified from pZR84 chromosomal DNA using primers 5-KpnI-GFP-U-pBAD18 and 3-XbaI-RodZ1-138-U-pBAD18.

The plasmid pZR105 (pBAD18, P_{BAD}::*gfp-rodZΔJM^{MalF13}*, *Amp^R*) was constructed by ligation of an KpnI/XbaI digested DNA fragment containing *gfp-rodZΔJM^{MalF13}* into pBAD18 digested with the same enzymes. The DNA fragment was amplified from pZR88 chromosomal DNA using primers 5-KpnI-RodZ1-155-U-PBAD18 and 3-XbaI-RodZ-U- pBAD18.

The plasmid pZR106 (pBAD18, P_{BAD}::*gfp-rodZΔJM$^{(2xMalF13)}$*, *Amp^R*) was constructed by ligation of an KpnI/XbaI digested DNA fragment containing *gfp-rodZΔJM$^{(2xMalF13)}$* into pBAD18 digested with the same enzymes. The DNA fragment was amplified from pZR89 chromosomal DNA using primers 5-KpnI-RodZ1-155-U-PBAD18 and 3-XbaI-RodZ-U- pBAD18. Derivatives of pZR106 carrying different alleles of *rodZΔJM$^{(2xMalF13)}$*

were obtained by site-directed mutagenesis using primer pairs listed in Supplementary Table S4.

The plasmid pZR130 (pMB1, *bla* P_{lac}::*t18-mreC*$^{33\text{-}367}$) was constructed by ligation of an XbaI/EcoRI digested DNA fragment containing *mreC*$^{33\text{-}367}$ into pUT18C (pMB1, *bla* P_{lac}::*t18-)* digested with the same enzymes. The DNA fragment was amplified from W3110 chromosomal DNA using primers 5-XbaI-MreC$^{33\text{-}367}$-T25C and 3-EcoRI-mreC$^{33\text{-}367}$-T25C. Derivatives of pZR130 carrying different alleles of *mreC* were obtained by site-directed mutagenesis using primer pairs listed in Supplementary Table S4.

The plasmid pZR131 (pACYC184, *Kan* P_{lac}::*t25-mreC*$^{33\text{-}367}$) was constructed by ligation of an XbaI/EcoRI digested DNA fragment containing *mreC*$^{33\text{-}367}$ into pKT25 (pACYC184, *Kan* P_{lac}*::t25-)* digested with the same enzymes. The DNA fragment was amplified from W3110 chromosomal DNA using primers 5-XbaI-MreC33-367-T25C and 3-EcoRI-mreC33-367-T25C. Derivatives of pZR131 carrying different alleles of *mreC*$^{33\text{-}367}$ were obtained by site-directed mutagenesis using primer pairs listed in Supplementary Table S4.

The plasmid pZR170 (pDSW209, *bla* P_{206}::*gfp-mreC*) was constructed by ligation of an EcoRI/HindIII digested DNA fragment containing *mreC* into pDSW209 digested with the same enzymes. The DNA fragment was amplified from W3110 chromosomal DNA using primers 5-EcoRI-MreC-GFP and 3-mreC-HindIII. Derivatives of pZR170 carrying different alleles of *mreC* were obtained by site-directed mutagenesis using primer pairs listed in Supplementary Table S4.

The plasmid pZR170-1-278 (pDSW209, *bla* P_{206}::*gfp-mreC*$^{1\text{-}278}$*)* as constructed by ligation of an EcoRI/HindIII digested DNA fragment containing *mreC*$^{1\text{-}278}$ into pDSW209 digested with the same enzymes. The DNA fragment was amplified from plasmid pSD315 using primers 5-EcoRI-MreC-GFP and 3-MreC-278-HindIII. Derivatives of pZR170-1-278 carrying different alleles of *mreC*$^{1\text{-}278}$ were obtained by site-directed mutagenesis using primer pairs listed in Supplementary Table S4.

The plasmid pZR170-1-305 (pDSW209, *bla* P_{206}::*gfp-mreC*$^{1\text{-}305}$) was constructed by ligation of an EcoRI/HindIII digested DNA fragment containing *mreC*$^{1\text{-}305}$ into pDSW209 digested with the same enzymes. The DNA fragment was amplified from plasmid pSD315 using primers 5-EcoRI-MreC-GFP and 3-MreC-305-HindIII. Derivatives of pZR170-1-305 carrying different alleles of *mreC*$^{1\text{-}305}$ were obtained by site-directed mutagenesis using primer pairs listed in Supplementary Table S4.

The plasmid pZR194 (pDSW209, *bla* P_{206}::*gfp-mreC-mreD*) was constructed by ligation of an EcoRI/HindIII digested DNA fragment containing *mreC-mreD* into pDSW209 digested with the same enzymes. The DNA fragment was amplified from W3110 chromosomal DNA using primers 5-EcoRI-MreC-GFP and 3-mreD-HindIII. Derivatives of pZR194 carrying different alleles of *mreC* were obtained by site-directed mutagenesis using primer pairs listed in Supplementary Table S4.

Plasmid pZR197 was obtained by site-directed mutagenesis of plasmid pZR194 using primers 5-MreC-C199S and 3-MreC-C199S.

The plasmid pZR210 (pDSW210, *bla* P_{206}::*flag-mreC*) was constructed by ligation of an XbaI/HindIII digested DNA fragment containing *flag-mreC* into pDSW210 digested with the same enzymes. The DNA fragment was amplified from plasmid pSD315 using primers 5-XbaI-Flag-MreC and 3-mreC-HindIII.

The plasmid pZR211 (pDSW210, *bla* P_{206}::*flag-mreC-mreD*) was constructed by ligation of an XbaI/HindIII digested DNA fragment containing *flag-mreC-mreD* into pDSW210 digested with the same enzymes. The DNA fragment was amplified from plasmid pSD315 using primers 5-XbaI-Flag-MreC and 3-mreD-HindIII.

The plasmid pZR212 (pDSW210, *bla* P_{206}::*flag-mreC*^{C175S,C199S}*-mreD*) was constructed by ligation of an XbaI/HindIII digested DNA fragment containing *flag-mreC*^{C175S,C199S}*-mreD* into pDSW210 digested with the same enzymes. The DNA fragment was amplified from plasmid pZR197 using primers 5-XbaI-Flag-MreC and 3-mreD-HindIII. Derivatives of pZR212 carrying different alleles of *mreC* were obtained by site-directed mutagenesis using primer pairs listed in Supplementary Table S4.

The plasmid pZR269 (pDSW209, *bla* P_{206}::*gfp-rodZ*$^{1-155}$*-flag-mreC*) was constructed by ligation of an XbaI/HindIII digested DNA fragment containing *flag-mreC* fragment into pZR84 digested with the same enzymes. The DNA fragment was amplified from pZR210 DNA using primers 5-XbaI-Flag-MreC and 3-MreC-HindIII.

The plasmid pZR270 (pDSW209, *bla* P_{206}::*gfp-rodZ*$^{1-138}$*-flag-mreC*) was constructed by ligation of an XbaI/HindIII digested DNA fragment containing *flag-mreC* fragment into pZR83 digested with the same enzymes. The DNA fragment was amplified from pZR210 DNA using primers 5-XbaI-Flag-MreC and 3-MreC-HindIII.

The plasmid pZR302 (pDSW210, *bla* P_{206}::*flag-linker-pbp2-gfp-mreC*) was constructed by ligation of an XbaI/HindIII digested DNA fragment containing *flag-linker-pbp2-gfp-mreC* into pDSW210 digested with the same enzymes. The *flag-linker-pbp2-gfp-mreC* DNA fragment was obtained by overlap extension PCR. Two DNA fragments containing *flag-linker-pbp2* and *gfp-mreC* were amplified from pZR293 and pZR170 using primer pairs 5-XbaI-FLAG-L-PBP2 / 3-GFP-PBP2 and 5-PBP2-GFP/3-HindIII-MreC-U. Derivatives of pZR302 carrying different alleles of *mreC* were obtained by site-directed mutagenesis using primer pairs listed in Supplementary Table S4.

The plasmid pZR303 (pDSW210, *bla* P_{206}::*flag-linker-pbp2-gfp-mreC*$^{1-305}$) was constructed by ligation of an XbaI/HindIII digested DNA fragment containing *flag-linker-pbp2-gfp-mreC(1-305)* into pDSW210 digested with the same enzymes. The *flag-linker-pbp2-gfp-mreC*$^{1-305}$ DNA fragment was obtained by overlap extension PCR. Two DNA fragments containing *flag-linker-pbp2* and *gfp-mreC*$^{1-305}$ were amplified from pZR293 and pZR170 using primer pairs 5-XbaI-FLAG-L-PBP2 / 3-GFP-PBP2 and 5-PBP2-GFP / 3-MreC-305-HindIII-U. Derivatives of pZR303 carrying different alleles of *mreC*$^{1-305}$ were obtained by site-directed mutagenesis using primer pairs listed in Table S4.

The plasmid pZR304 (pDSW210, *bla* P_{206}::*flag-linker-pbp2-gfp-mreC*$^{1-278}$) was constructed by ligation of an XbaI/HindIII digested DNA fragment containing *flag-linker-pbp2-gfp-mreC*$^{1-278}$ into pDSW210 digested with the same enzymes. The *flag-linker-*

pbp2-gfp-mreC$^{1-278}$ DNA fragment was obtained by overlap extension PCR. Two DNA fragments containing *flag-linker-pbp2* and *gfp-mreC*$^{1-278}$ were amplified from pZR293 and pZR170 using primer pairs 5-XbaI-FLAG-L-PBP2/3-GFP-PBP2 and 5-PBP2-GFP/3-MreC1-278-HindIII-U. Derivatives of pZR304 carrying different alleles of *mreC*$^{1-278}$ were obtained by site-directed mutagenesis using primer pairs listed in Table S4.

S1 table. Mutations in the plasmids providing resistance to A22.

Plasmid	Mutations	Resistance to A22 (10 μg/mL)
M1	mreC-P150L (CCG/CCA); mreC-V164V (GTC/GTT); mreC-R264H (CGT/CAT); mreD-W38stop (TGG/TAG); mreD-L68P (CTG/CCG); mreD-V118A (GTT/GCT)	Yes
M2	mreC-G259V (GGG/GTG); mreD-L50F (TTG/TTC)	Yes
M3	mreC-R264H (CGT/CAT); mreC-A289A (GCT/GCA); mreC-R362H (CGT/CAT); mreD-G63C (GGT/TGT); mreD-P132S (CCG/TCG)	Yes
M4	mreC-I183F (ATC/TTC); mreD-V11V (GTA/GTT); mreD-G59V (GGT/GTT)	Yes
M5	mreC-P150L (CCG/CTG); mreC-P279T (CCG/ACG)	Yes
M6	mreC-T40A (ACT/GCT); mreC-L173L (CTG/TTG); mreC-L205F (CTT/TTT); mreC-A210V (GCT/GTG); mreC-F229I (TTC/ATC); mreC-S239Y (TCT/TAT); mreC-V300A (GTA/GCA); mreC-A318T (ATC/ACC)	Yes
M7	mreC-A194V (GCA/GTA); mreC-E231V (GAA/GTA); mreC-L267V (CTG/GTG); mreD-F119L (TTC/TTA)	Yes
M8	mreC-S69S (TCG/TCA); mreC-P182L (CCA/CTA); mreD-I148F (ATT/TTT)	Yes
M9	mreC-S153R (AGC/AGA)	Yes
M10	mreC-R57L (CGT/CTT); mreC-D133H (GAT/CAT); mreC-T222A (ACT/GCT); mreC-A334V (GCG/GTG)	Yes
M11	mreC-E100D (GAG/GAT); mreC-Q117R (CAG/CGG); mreC-R274H (CGT/CAT); mreD-L112L (CTG/CTA)	Yes
M12	mreC-T63T (ACG/ACA); mreC-D154N (GAC/AAC), mreC-D273E (GAT/GAA)	Yes
M13	mreC-P150L (CCG/CTG); mreC-I183V (ATC/GTC); mreD-D73D (ACG/ACA)	Yes
M14	mreC-Q184L (CAG/CTG); mreD-P51T (CCT/ACT)	Yes
M15	mreC-A68T (GCC/ACC); mreC-P150L (CCG/CTG); mreC-A363A (GCG/GCT); mreC-G366E (GGG/GAG)	Yes
M16	mreC-E231K (GAA/AAA); mreC-Q299Stop (CAG/TAG)	Yes

S2 Table. Bacterial strains used in this study

Strain	Genotype	Source
BTH101	*cya-99 araD139 galE15 galK16 rpsL1 hsdR2 mcrA1 mcrB1*	(4)
JS238	MC1061*, malP* p*::lacIQ srlC::Tn10 recA1*	(5)
TB28	MG1655, Δ*lacIZYA*::*frt*	(6)
P3988	FB38(λTB59) [TB28, *mrdAB<>aph* (*attλ bla lacI*^{q} P$_{lac}$::*mrdAB*)]	(7)
P4206	FB30/pFB174 [TB28, *MreBCD<>aph* / pBAD33, P$_{BAD}$::*mreBCD*-le *cat* araC]	(7)
P5439	FB60/pFB290 [TB28, *rodZ<>aph* **/** ColE1, *bla lacI*^{q} P$_{lac}$::*rodZ*]	(7, 8)
SD464	TB28, *mreCD<>aph* / pSD352 (P$_{BAD}$::*mreCD)*	This study
SD475	TB28, *yhdE*::*cat*	This study
SD495	TB28, *yhdE*::*cat mreC*^{E100D}	This study
SD496	TB28, *yhdE*::*cat mreC*^{P150L}	This study
SD497	TB28, *yhdE*::*cat mreC*^{S153R}	This study
SD498	TB28, *yhdE*::*cat mreC*^{D154N}	This study
SD499	TB28, *yhdE*::*cat mreC*^{P182L}	This study
SD501	TB28, *yhdE*::*cat mreC*^{Q184L}	This study
SD502	TB28, *yhdE*::*cat mreC*^{T222A}	This study
SD503	TB28, *yhdE*::*cat mreC*^{E231K}	This study
SD504	TB28, *yhdE*::*cat mreC*^{R264H}	This study
SD541	TB28*, yhdE<>cat ΔrodZ*::*aph*	This study
SD542	TB28*, yhdE<>cat mreC*^{E100D}, *ΔrodZ*::*aph*	This study
SD543	TB28*, yhdE<>cat mreC*^{P150L}, *ΔrodZ*::*aph*	This study
SD544	TB28*, yhdE<>cat mreC*^{S153R}, *ΔrodZ*::*aph*	This study
SD545	TB28*, yhdE<>cat mreC*^{D154N}, *ΔrodZ*::*aph*	This study
SD546	TB28*, yhdE<>cat mreC*^{P182L}, *ΔrodZ*::*aph*	This study
SD547	TB28*, yhdE<>cat mreC*^{Q184L}, *ΔrodZ*::*aph*	This study
SD548	TB28*, yhdE<>cat mreC*^{T222A}, *ΔrodZ*::*aph*	This study
SD549	TB28*, yhdE<>cat mreC*^{E231K}, *ΔrodZ*::*aph*	This study
SD550	TB28*, yhdE<>cat mreC*^{R264H}, *ΔrodZ*::*aph*	This study
RZ7	TB28, P$_{lac}$::*mreB'-mNeonGreen-'mreB*	This study
RZ8	TB28, Δ*yhdE<>frt*	This study
RZ9	TB28, Δ*yhdE<>frt mreC*^{E100D}	This study
RZ10	TB28, Δ*yhdE<>frt mreC*^{P150L}	This study
RZ11	TB28, Δ*yhdE<>frt mreC*^{P182L}	This study
RZ12	TB28, Δ*yhdE<>frt mreC*^{T222A}	This study
RZ13	TB28, Δ*yhdE<>frt mreC*^{E231K}	This study
RZ14	TB28, Δ*yhdE<>frt mreC*^{R264H}	This study
RZ15	TB28, Δ*yhdE<>frt, attλ,* P$_{lac}$::*mreB'-mNeonGreen-'mreB*	This study
RZ16	TB28, Δ*yhdE<>frt mreC*^{E100D}*, attλ,* P$_{lac}$::*mreB'-mNeonGreen-'mreB*	This study

RZ17	TB28 , Δ*yhdE*<>*frt* $mreC^{P150L}$, *attλ,* P_{lac}::*mreB'*-*mNe nGreen-'mreB*	This study
RZ18	TB28 , Δ*yhdE*<>*frt* $mreC^{P182L}$, *attλ,* P_{lac}::*mreB'*-*mNe nGreen-'mreB*	This study
RZ19	TB28 , Δ*yhdE*<>*frt* $mreC^{T222A}$, *attλ,* P_{lac}::*mreB'*-*mNe nGreen-'mreB*	This study
RZ20	TB28 , Δ*yhdE*<>*frt* $mreC^{E231K}$, *attλ,* P_{lac}::*mreB'*-*mNe nGreen-'mreB*	This study
RZ21	TB28 , Δ*yhdE*<>*frt* $mreC^{R264H}$, *attλ,* P_{lac}::*mreB'*-*mNe nGreen-'mreB*	This study

S3 Table. Plasmids used in this study

Plasmid	Genotype	Source
pDSW209	pBR322 pDSW206-*gfp-MCS*, Amp^R	(9)
pDSW210	pBR322 pDSW206-*MCS-gfp*, Amp^R	(9)
pHC892	ColE1, P_{lac}:: *mreB'-mNeonGreen-'mreB* Amp^R	(10)
pHC897	R6K, *attλ cat lacI*^{q} P_{lac}::*mreB'-mNeonGreen-'mreB*	(10)
P_{int}λ	pSC101^{ts}, *bla cI875 repA(Ts)* P_R::*intλ*	(3)
pKT25	pACYC184, *kan* P_{lac}::*t25-*	(4)
pUT18C	pMB1, *bla* P_{lac}::*t18-*	(4)
pGB2	pSC101, Spc^R	(11)
pHGB2	pSC101^{ts}, Spc^R	(11)
pCP20	pSC101^{ts}, *cI857 repA*^{ts}*, flp recombinase*, Amp^R	(12)
pBAD33	p15A, P_{bad}::*mcs*, Cam^R	(13)
pBAD18	M13, P_{ara}::*MCS-*, Amp^R	(13)
pSD312	p15A pBAD33, P_{BAD}::*pbp2-rodA*, Cam^R	This study
pSD315	pBR322 pDSW210, *bla* P_{206}::*mreC-mreD*	This study
pSD315-E100D	pBR322 pDSW210, *bla* P_{206}::*mreC*^{E100D}*-mreD*	This study
pSD315-P150L	pBR322 pDSW210, *bla* P_{206}::*mreC*^{P150L}*-mreD*	This study
pSD315-S153R	pBR322 pDSW210, *bla* P_{206}::*mreC*^{S153R}*-mreD*	This study
pSD315-D154N	pBR322 pDSW210, *bla* P_{206}::*mreC*^{D154N}*-mreD*	This study
pSD315-P182L	pBR322 pDSW210, *bla* P_{206}::*mreC*^{P182L}*-mreD*	This study
pSD315-I183F	pBR322 pDSW210, *bla* P_{206}::*mreC*^{I183F}*-mreD*	This study
pSD315-Q184L	pBR322 pDSW210, *bla* P_{206}::*mreC*^{Q184L}*-mreD*	This study
pSD315-T222A	pBR322 pDSW210, *bla* P_{206}::*mreC*^{T222A}*-mreD*	This study
pSD315-E231K	pBR322 pDSW210, *bla* P_{206}::*mreC*^{E231K}*-mreD*	This study
pSD315-E231V	pBR322 pDSW210, *bla* P_{206}::*mreC*^{E231V}*-mreD*	This study
pSD315-R264H	pBR322 pDSW210, *bla* P_{206}::*mreC*^{R264H}*-mreD*	This study
pSD315-G156D	pBR322 pDSW210, *bla* P_{206}::*mreC*^{G156D}*-mreD*	This study
pSD315-R292H	pBR322 pDSW210, *bla* P_{206}::*mreC*^{R292H}*-mreD*	This study
pSD315-G259V	pBR322 pDSW210, *bla* P_{206}::*mreC*^{G259V}*-mreD*	This study
pSD315-L50F	pBR322 pDSW210, *bla* P_{206}::*mreC-mreD*^{L50F}	This study
pSD315-G259V&L50F	pBR322 pDSW210, *bla* P_{206}::*mreC*^{G259V}*-mreD*^{L50F}	This study
pSD315-E100D&G156D	pBR322 pDSW210, *bla* P_{206}::*mreC*^{E100D,G156D}*-mreD*	This study
pSD315-P150L&G156D	pBR322 pDSW210, *bla* P_{206}::*mreC*^{P150L,G156D}*-mreD*	This study
pSD315-P182L&G156D	pBR322 pDSW210, *bla* P_{206}::*mreC*^{G156D,P182L} *-mreD*	This study
pSD315-T222A&G156D	pBR322 pDSW210, *bla* P_{206}::*mreC*^{G156D,T222A}*-mreD*	This study
pSD315-E231K&G156D	pBR322 pDSW210, *bla* P_{206}::*mreC*^{G156D,E231K}*-mreD*	This study

pSD315-R264H&G156D	pBR322 pDSW210, *bla* P_{206}::*mreC*^{G156D,R264H}-*mreD*	This study
pSD315-E100D&R292H	pBR322 pDSW210, *bla* P_{206}::*mreC*^{E100D,R292H}-*mreD*	This study
pSD315-P150L&R292H	pBR322 pDSW210, *bla* P_{206}::*mreC*^{P150L,R292H}-*mreD*	This study
pSD315-P182L&R292H	pBR322 pDSW210, *bla* P_{206}::*mreC*^{P182L,R292H}-*mreD*	This study
pSD315-T222A&R292H	pBR322 pDSW210, *bla* P_{206}::*mreC*$^{T222A,R292H-}$*mreD*	This study
pSD315-E231K&R292H	pBR322 pDSW210, *bla* P_{206}::*mreC*^{E231K,R292H}-*mreD*	This study
pSD315-R264H&R292H	pBR322 pDSW210, *bla* P_{206}::*mreC*^{R264H,R292H}-*mreD*	This study
pSD315-AL-DD	pBR322 pDSW210, *bla* P_{206}::*mreC*^{A79D,L80D}-*mreD*	This study
pSD315-1-278	pBR322 pDSW210, *bla* P_{206}::*mreC(1-278)-mreD*	This study
pSD315-1-305	pBR322 pDSW210, *bla* P_{206}::*mreC(1-305)-mreD*	This study
pSD315-1-278-E100D	pBR322 pDSW210, *bla* P_{206}::*mreC(1-278)*^{E100D}-*mreD*	This study
pSD315-1-278-P150L	pBR322 pDSW210, *bla* P_{206}::*mreC(1-278)*^{P150L}-*mreD*	This study
pSD315-1-278-T222A	pBR322 pDSW210, *bla* P_{206}::*mreC(1-278)*^{T222A}-*mreD*	This study
pSD315-1-278-E231K	pBR322 pDSW210, *bla* P_{206}::*mreC(1-278)*^{E231K}-*mreD*	This study
pSD315-1-278-R264H	pBR322 pDSW210, *bla* P_{206}::*mreC(1-278)*^{R264H}-*mreD*	This study
pSD352	p15A pBAD33, P_{BAD}::*mreC-mreD*, *Cam*^{R}	This study
pZR4	pSC101^{ts}, *mreC-mreD, Spc*^{R}	This study
pZR4-E100D	pSC101^{ts}, *mreC*^{E100D}-*mreD*, *Spc*^{R}	This study
pZR4-P150L	pSC101^{ts}, *mreC*^{P150L}-*mreD*, *Spc*^{R}	This study
pZR4-S153R	pSC101^{ts}, *mreC*^{S153R}-*mreD*, *Spc*^{R}	This study
pZR4-D154N	pSC101^{ts}, *mreC*^{D154N}-*mreD*, *Spc*^{R}	This study
pZR4-P182L	pSC101^{ts}, *mreC*^{P182L}-*mreD*, *Spc*^{R}	This study
pZR4-Q184L	pSC101^{ts}, *mreC*^{Q184L}-*mreD*, *Spc*^{R}	This study
pZR4-T222A	pSC101^{ts}, *mreC*^{T222A}-*mreD*, *Spc*^{R}	This study
pZR4-E231K	pSC101^{ts}, *mreC*^{E231K}-*mreD*, *Spc*^{R}	This study
pZR4-R264H	pSC101^{ts}, *mreC*^{R264H}-*mreD*, *Spc*^{R}	This study
pZR7	pMB1, *bla* P_{lac}::*t18-mreC*	This study
pZR7-AL-DD	pMB1, *bla* P_{lac}::*t18-mreC*^{A79D,L80D}	This study
pZR7-1-278	pMB1, *bla* P_{lac}::*t18-mreC(1-278)*	This study
pZR7-1-305	pMB1, *bla* P_{lac}::*t18-mreC(1-305)*	This study
pZR7-1-305-G156D	pMB1, *bla* P_{lac}::*t18-mreC(1-305)*^{G156D}	This study
pZR7-1-305-R292H	pMB1, *bla* P_{lac}::*t18-mreC(1-305)*^{R292H}	This study

pZR7-1-278-E100D	pMB1, *bla* P_{lac}::*t18-mreC(1-278)*^{E100D}	This study
pZR7-1-278-P150L	pMB1, *bla* P_{lac}::*t18-mreC(1-278)*^{P150L}	This study
pZR7-1-278-P182L	pMB1, *bla* P_{lac}::*t18-mreC(1-278)*^{P182L}	This study
pZR7-1-278-Q184L	pMB1, *bla* P_{lac}::*t18-mreC(1-278)*^{Q184L}	This study
pZR7-1-278-T222A	pMB1, *bla* P_{lac}::*t18-mreC(1-278)*^{T222A}	This study
pZR7-1-278-E231K	pMB1, *bla* P_{lac}::*t18-mreC(1-278)*^{E231K}	This study
pZR7-1-278-R264H	pMB1, *bla* P_{lac}::*t18-mreC(1-278)*^{R264H}	This study
pZR8	pACYC184, *kan* P_{lac}::*t25-mreC*	This study
pZR8-AL-DD	pACYC184, *kan* P_{lac}::*t25-mreC*^{A79D,L80D}	This study
pZR10	pACYC184, *kan* P_{lac}::*t25-mreD*	This study
pZR12	pACYC184, *kan* P_{lac}::*t25-pbp2*	This study
pZR14	pACYC184, *kan* P_{lac}::*t25-rodA*	This study
pZR17	pMB1, *bla* P_{lac}::*t18-rodZ*	This study
pZR17-1-155	pMB1, *bla* P_{lac}::*t18-rodZ(1-155)*	This study
pZR17-1-155-QE-KK	pMB1, *bla* P_{lac}::*t18-rodZ(1-155)* ^{Q140K,E141K}	This study
pZR18	pACYC184, *kan* P_{lac}::*t25-rodZ*	This study
pZR18-1-155	pACYC184, *kan* P_{lac}::*t25-rodZ(1-155)*	This study
pZR26	ColE1, P_{lac}::*mreB'-t25-mreB'*, *Amp*^{R}	This study
pZR28	pACYC184, *kan* P_{lac}::*t25- mreB'-t25c-mreB'*	This study
pZR82	pBR322 pDSW209, *bla* P_{206}::*gfp-rodZ*	This study
pZR83	pBR322 pDSW209, *bla* P_{206}::*gfp-rodZ(1-138)*	This study
pZR84	pBR322 pDSW209, *bla* P_{206}::*gfp-rodZ(1-155)*	This study
pZR84	pBR322 pDSW209, *bla* P_{206}::*gfp-rodZ(1-155)*	This study
pZR86	pBR322 pDSW209, *bla* P_{206}::*gfp-rodZ(1-155) ΔJM*^{MalF13}	This study
pZR87	pBR322 pDSW209, *bla* P_{206}::*gfp-rodZ(1-155) ΔJM*$^{(2×MalF13)}$	This study
pZR88	pBR322 pDSW209, *bla* P_{206}::*gfp-rodZΔJM*^{MalF13}	This study
pZR89	pBR322 pDSW209, *bla* P_{206}::*gfp-rodZ ΔJM*$^{(2×MalF13)}$	This study
pZR95	M13 pBAD18, P_{BAD}::*rodZ*, *Amp*^{R}	This study
pZR96	M13 pBAD18, P_{BAD}::*rodZ(1-155)*, *Amp*^{R}	This study
pZR96-QE-KK	M13 pBAD18, P_{BAD}::*rodZ(1-155)* ^{Q140K,E141K}, *Amp*^{R}	This study
pZR97	M13 pBAD18, P_{BAD}::*rodZ(1-138)*, *Amp*^{R}	This study
pZR102	M13 pBAD18, P_{BAD}::*gfp-rodZ*, *Amp*^{R}	This study
pZR102-F60A	M13 pBAD18, P_{BAD}::*gfp-rodZ*^{F60A}, *Amp*^{R}	This study
pZR102-QE	M13 pBAD18, P_{BAD}::*gfp-rodZ*$^{QE-KK}$, *Amp*^{R}	This study
pZR102-F60A&QE	M13 pBAD18, P_{BAD}::*gfp-rodZ*$^{F60A,QE-KK}$, *Amp*^{R}	This study
pZR103	M13 pBAD18, P_{BAD}::*gfp-rodZ*$^{1-155}$, *Amp*^{R}	This study
pZR104	M13 pBAD18, P_{BAD}::*gfp-rodZ*$^{1-138}$, *Amp*^{R}	This study
pZR105	M13 pBAD18, P_{BAD}::*gfp-rodZΔJM*^{MalF13}, *Amp*^{R}	This study

pZR106	M13 pBAD18, P_{BAD}::*gfp-rodZΔJM*$^{(2×MalF13)}$, *Amp*^{R}	This study
pZR106-F60A	M13 pBAD18, P_{BAD}::*gfp-rodZΔJM*$^{(2×MalF13),F60A}$, *Amp*^{R}	This study
pZR106-QE	M13 pBAD18, P_{BAD}::*gfp-rodZΔJM*$^{(2×MalF13),QE-KK}$, *Amp*^{R}	This study
pZR107	M13 pBAD18, P_{BAD}::*gfp-rodZΔHTH*, *Amp*^{R}	This study
pZR130	pMB1, *bla* P_{lac}::*t18-mreC(33-367)*	This study
pZR131	pACYC184, *kan* P_{lac}::*t25-mreC(33-367)*	This study
pZR130-E100D	pMB1, *bla* P_{lac}::*t18-mreC(33-367)*^{E100D}	This study
pZR131-E100D	pACYC184, *kan* P_{lac}::*t25-mreC(33-367)*^{E100D}	This study
pZR130-P150L	pMB1, *bla* P_{lac}::*t18-mreC(33-367)*^{P150L}	This study
pZR131-P150L	pACYC184, *kan* P_{lac}::*t25-mreC(33-367)*^{P150L}	This study
pZR130-S153R	pMB1, *bla* P_{lac}::*t18-mreC(33-367)*^{S153R}	This study
pZR131-S153R	pACYC184, *kan* P_{lac}::*t25-mreC(33-367)*^{S153R}	This study
pZR130-D154N	pMB1, *bla* P_{lac}::*t18-mreC(33-367)*^{D154N}	This study
pZR131-D154N	pACYC184, *kan* P_{lac}::*t25-mreC(33-367)*^{D154N}	This study
pZR130-P182L	pMB1, *bla* P_{lac}::*t18-mreC(33-367)*^{P182L}	This study
pZR131-P182L	pACYC184, *kan* P_{lac}::*t25-mreC(33-367)*^{P182L}	This study
pZR130-Q184L	pMB1, *bla* P_{lac}::*t18-mreC(33-367)*^{Q184L}	This study
pZR131-Q184L	pACYC184, *kan* P_{lac}::*t25-mreC(33-367)*^{Q184L}	This study
pZR130-T222A	pMB1, *bla* P_{lac}::*t18-mreC(33-367)*^{T222A}	This study
pZR131-T222A	pACYC184, *kan* P_{lac}::*t25-mreC(33-367)*^{T222A}	This study
pZR130-E231K	pMB1, *bla* P_{lac}::*t18-mreC(33-367)*^{E231K}	This study
pZR131-E231K	pACYC184, *kan* P_{lac}::*t25-mreC(33-367)*^{E231K}	This study
pZR130-R264H	pMB1, *bla* P_{lac}::*t18-mreC(33-367)*^{R264H}	This study
pZR131-R264H	pACYC184, *kan* P_{lac}::*t25-mreC(33-367)*^{R264H}	This study
pZR130-G156D	pMB1, *bla* P_{lac}::*t18-mreC(33-367)*^{G156D}	This study
pZR131-G156D	pACYC184, *kan* P_{lac}::*t25-mreC(33-367)*^{G156D}	This study
pZR130-R292H	pMB1, *bla* P_{lac}::*t18-mreC(33-367)*^{R292H}	This study
pZR131-R292H	pACYC184, *kan* P_{lac}::*t25-mreC(33-367)*^{R292H}	This study
pZR170	pBR322 pDSW209, *bla* P_{206}::*gfp-mreC-mreD*	This study
pZR170-1-278	pBR322 pDSW209, *bla* P_{206}::*gfp-mreC(1-278)-mreD*	This study
pZR170-1-278^{E100D}	pBR322 pDSW209, *bla* P_{206}::*gfp-mreC(1-278)* ^{E100D} *-mreD*	This study
pZR170-1-278^{P150L}	pBR322 pDSW209, *bla* P_{206}::*gfp-mreC(1-278)* ^{P150L} *-mreD*	This study
pZR170-1-278^{P182L}	pBR322 pDSW209, *bla* P_{206}::*gfp-mreC(1-278)* ^{P182L} *-mreD*	This study
pZR170-1-278^{T222A}	pBR322 pDSW209, *bla* P_{206}::*gfp-mreC(1-278)* ^{T222A}*-mreD*	This study
pZR170-1-278^{E231K}	pBR322 pDSW209, *bla* P_{206}::*gfp-mreC(1-278)* ^{E231K}*-mreD*	This study

pZR170-1-278^{R264H}	pBR322 pDSW209, *bla* P_{206}::*gfp-mreC(1-278)*^{R264H}*-mreD*	This study
pZR170-1-305	pBR322 pDSW209, *bla* P_{206}::*gfp-mreC(1-305)-mreD*	This study
pZR170-1-305-G156D	pBR322 pDSW209, *bla* P_{206}::*gfp-mreC(1-305)*^{G156D}*-mreD*	This study
pZR170-1-305-R292H	pBR322 pDSW209, *bla* P_{206}::*gfp-mreC(1-305)*^{R292H}*-mreD*	This study
pZR170-AL-DD	pBR322 pDSW209, *bla* P_{206}::*gfp-mreC*^{A79D,L80D}	This study
pZR197	pBR322 pDSW210, *bla* P_{206}::*gfp-mreC*^{C175S,C199S}*-mreD*	This study
pZR210	pBR322 pDSW210, *bla* P_{206}::*flag-mreC*	This study
pZR211	pBR322 pDSW210, *bla* P_{206}::*flag-mreC-mreD*	This study
pZR212	pBR322 pDSW210, *bla* P_{206}::*flag-mreC*^{C175S,C199S}*-mreD*	This study
pZR212-L108C	pBR322 pDSW210, *bla* P_{206}::*flag-mreC*^{L108C,C175S,C199S} *-mreD*	This study
pZR212-Q122C	pBR322 pDSW210, *bla* P_{206}::*flag-mreC*^{Q122C,C175S,C199S} *-mreD*	This study
pZR212-L108C&E100D	pBR322 pDSW210, *bla* P_{206}::*flag-mreC*^{E100D,L108C,C175S,C199S} *-mreD*	This study
pZR212-L108C&P150L	pBR322 pDSW210, *bla* P_{206}::*flag-mreC*^{L108C,P150L,C175S,C199S}*-mreD*	This study
pZR212-L108C&P182L	pBR322 pDSW210, *bla* P_{206}::*flag-mreC* ^{L108C,P182L,C175S,C199S} *-mreD*	This study
pZR212-L108C&T222A	pBR322 pDSW210, *bla* P_{206}::*flag-mreC*^{L108C,C175S,C199S,T222A}*-mreD*	This study
pZR212-L108C&E231K	pBR322 pDSW210, *bla* P_{206}::*flag-mreC*^{L108C,C175S,C199S,E231K}*-mreD*	This study
pZR212-L108C&R264H	pBR322 pDSW210, *bla* P_{206}::*flag-mreC*^{L108C,C175S,C199S,R264H}*-mreD*	This study
pZR212-L108C&G156D	pBR322 pDSW210, *bla* P_{206}::*flag-mreC* $^{L108C,G156D\ C175S,C199S}$ *-mreD*	This study
pZR212-L108C&R292H	pBR322 pDSW210, *bla* P_{206}::*flag-mreC*^{L108C,C175S,C199S,R292H}*-mreD*	This study
pZR269	pBR322 pDSW209, *bla* P_{206}::*gfp-rodZ*$^{1-155}$*-flag-mreC*	This study
pZR270	pBR322 pDSW209, *bla* P_{206}::*gfp-rodZ*$^{1-138}$*-flag-mreC*	This study
pZR293	pBR322 pDSW210, *bla* P_{206}::*flag-linker-pbp2*	This study
pZR302	pBR322 pDSW210, *bla* P_{206}::*flag-linker-pbp2-gfp-mreC*	This study
pZR303	pBR322 pDSW210, *bla* P_{206}::*flag-linker-pbp2-gfp-mreC(1-305)*	This study

pZR303-G156D	pBR322 pDSW210, *bla* P_{206}::*flag-linker-pbp2-gfp-mreC(1-305)*^{G156D}	This study
pZR303-R292H	pBR322 pDSW210, *bla* P_{206}::*flag-linker-pbp2-gfp-mreC(1-305)*^{R292H}	This study
pZR304	pBR322 pDSW210, *bla* P_{206}::*flag-linker-pbp2-gfp-mreC(1-278)*	This study
pZR304-E231K	pBR322 pDSW210, *bla* P_{206}::*flag-linker-pbp2-gfp-mreC(1-278)*^{E231K}	This study
pZR304-R264H	pBR322 pDSW210, *bla* P_{206}::*flag-linker-pbp2-gfp-mreC(1-278)*^{R264H}	This study

S4 Table. Primers used in this study

Primer name	Sequence
5-rodZ-seqF	TTGTCACGAGCACACAGACGGTCT
3-rodZ-seqR	ACTGTACGGCGATGGGAGCACCAT
5-mreC-SeqF	TCGGATGCAGGCAGGGGAAGTG
3-mreD-seqR	TGCTGCGCGCTCTCCTGCGG
5-HindIII-MreC	gcAAGCTTATGAAGCCAATTTTTAGCCG
3-EcoRI-MreD	gcGAATTCTTATTGCACTGCAAACTGCT
5-EcoRI-mreC	GCGAATTCGATACGAGAATACGCATAACTTATGAA
3-mreD-HindIII	GACTAAGCTTGTCATAGAAACCTTTATTGCACTG
mreC-E100D-F	GGACAATACAAACAGGATAACGCGCGTCTGCGC
mreC-E100D-R	GCGCAGACGCGCGTTATCCTGTTTGTATTGTCC
mreC-P150L-F	GTTTATGAAGGCCAGCTGGTCATCAGCGACAAAG
mreC-P150L-R	CTTTGTCGCTGATGACCAGCTGGCCTTCATAAAC
mreC-S153R-F	GGCCAGCCGGTCATCAGAGACAAAGGTGTTGTT
mreC-S153R-R	AACAACACCTTTGTCTCTGATGACCGGCTGGCC
mreC-D154N-F	CAGCCGGTCATCAGCAACAAAGGTGTTGTTGGT
mreC-D154N-R	ACCAACAACACCTTTGTTGCTGATGACCGGCTG
mreC-G156D-F	GTCATCAGCGACAAAGATGTTGTTGGTCAGGTG
mreC-G156D-R	CACCTGACCAACAACATCTTTGTCGCTGATGAC
mreC-P182L-F	GCGACCCACGCGCTGCTAATCCAGGTGCTGCGC
mreC-P182L-R	GCGCAGCACCTGGATTAGCAGCGCGTGGGTCGC
mreC-I183F-F	ACCCACGCGCTGCCATTCCAGGTGCTGCGCAAC
mreC-I183F-R	GTTGCGCAGCACCTGGAATGGCAGCGCGTGGGT
mreC-E231K-F	GGCGGTCGTTTCCCGAAAGGCTATCCGGTCGCG
mreC-E231K-R	CGCGACCGGATAGCCTTTCGGGAAACGACCGCC
mreC-T222A-F	GGTGATGTGCTGGTGgCTTCCGGTCTGGGCGGTC
mreC-T222A-R	GACCGCCCAGACCGGAAGCCACCAGCACATCACC
mreC-R292H-F	CGTGTTGCTAATGAACATCTGATGCAGATGATG
mreC-R292H-R	CATCATCTGCATCAGACGTTCATTAGCAACACG
5-mreC-R264H	GGGCTGCAACGTTTGGatTATCTGCTGCTGCTG
3-mreC-R264H	CAGCAGCAGCAGATAatCCAAACGTTGCAGCCC
5-XbaI-mreC33-367-T25C	GCTCTAGAGGGGACGTTCAgtcaaaTCCGT
3-EcoRI-mreC33-367-T25C	CGGAATTCTCATTGCCCTCCCGGCGCACGCGC
T18C-out-R	TGTCTGTAAGCGGATGCCGG
T18C-in-F	GGCTATGTCTTCTACGAGAACCGTG
5-EcoRI-mreC-gfp	CCGAATTCAAGCCAATTTTTAGCCGTG
3-mreC-HindIII	CCAAGCTTCTACTATTGCCCTCCCGGCGCACGCG
3-mreC305-HindIII	GGCTACGATAGCTCGCCACTAGTCTGGCGAAGGCAA
3-mreC278-HindIII	CCAAGCTTCTAGTTAGCGCCGTTACGATCT
5-mreC-C199S	GCAGCCGGTAACGGTaGTACGGATGATTTGCAG
3-mreC-C199S	CTGCAAATCATCCGTACtACCGTTACCGGCTGC

5-mreC-C175S	CGCGTGCTGCTGATTAGTGATGCGACCCACGCG
3-mreC-C175S	CGCGTGGGTCGCATCACTAATCAGCAGCACGCG
5-mreC-L108C	CGTCTGCGCGAGCTGTGTGGTTCCCCGCTGCGT
3-mreC-L108C	ACGCAGCGGGGAACCACACAGCTCGCGCAGACG
5-mreC-Q122C	CAGAAAATGGTGACTTGTGTTATCTCCACGGTT
3-mreC-Q122C	AACCGTGGAGATAACACAAGTCACCATTTTCTG
3-mreC-C199S, T200C	AAGCTGCAAATCATCACAACTACCGTTACCGGCTGC
5-mreC-C199S, T200C	GCAGCCGGTAACGGTAGTTGTGATGATTTGCAGCTT
5-XbaI-flag-mreC	GCTCTAGAATGGATTACAAGGATGACGACGATAAGAAG CCAATTTTTAGCCGTGG
5-mreC-278-mreD	CGTAACGGCGCTAACTAGTGGCGAGCTATCGTAGCC
3-mreC-278/mreD	GGCTACGATAGCTCGCCACTAGTTAGCGCCGTTACG
5-mreC-305/mreD	TTGCCTTCGCCAGACTAGTGGCGAGCTATCGTAGCC
3-mreC-305/mreD	GGCTACGATAGCTCGCCACTAGTCTGGCGAAGGCAA
3-mreC-278-HindIII	CCAAGCTTCTAGTTAGCGCCGTTACGATCT
3-meC-305-HindIII	GGAAGCTTCTAGTCTGGCGAAGGCAATACC
T18C-XbaI-mreC	GCTCTAGAGAAGCCAATTTTTAGCC
mreC-KpnI-T18C	GGGGTACCCTATTGCCCTCCCGGC
3-mreC-1-278-KpnI	GCGGTACCTCAGTTAGCGCCGTTACGATCTGCC
3-mreC-1-305-KpnI	GCGGTACCCTAGTCTGGCGAAGGCAATACCTG
T25C-XbaI-mreD	GCTCTAGAGGCGAGCTATCGTAGCC
mreD-KpnI-T25C	GGGGTACCTTATTGCACTGCAAAC
T25-XbaI-pbp2	GCTCTAGAGAAACTACAGAACTCTTTT
pbp2-EcoRI-T25C	gcGAATTCTTAATGGTCCTCCGCTGC
T25C-XbaI-rodA	GCTCTAGAGACGGATAATCCGAATAA
rodA-KpnI-T25C	GGGGTACCTTACACGCTTTTCGACA
T25C-XbaI-rodZ	gCTCTAGAGAATACTGAAGCCACGCA
rodZ-KpnI-T25C	ggGGTACCTTACTGCGCCGGTGATTG
5-XbaI-mreC	GACTTCTAGATACGAGAATACGCATAACTT
5-XbaI-pbp2	GCTCTAGATTTGAGTAGAAAACGCAGC
5-KpnI-rodZ	GCGGTACCTCAATTCTCATTTAAACGTACC
3-rodZ-1-155-XbaI	GCTCTAGATTAGCTCAGTTCCGCCGAAGATTGATC
5-rodZ-F60A	GATCTTGCTTCAACAGCCCTGCGCGGATATATC
3-rodZ-F60A	GATATATCCGCGCAGGGCTGTTGAAGCAAGATC
5-kpnI-rodZΔHTH-gfp	CCGGTACCCTGGAAAAGCAGGCTCCACTTCGG
3-XbaI-rodZ	gcTCTAGATTACTGCGCCGGTGATT
5-kpnI-gfp-rodZ	ggGGTACCAATACTGAAGCCACGCACGACC
3-XbaI-RodZ-U-pBAD18	TTGCATGCCTGCAGGTCGACTCTAGATTACTGCGCCG GTGATTGTT
5-KpnI-GFP-U-pBAD18	GGGCTAGCGAATTCGAGCTCGGTACCATGAGTAAAGG AGAAGAACT

3-rodZ-1-138-XbaI	GCTCTAGATTAAGCTTTGCGGTCTTGCCA
3-rodZ155-KpnI	CCGGTACCTTAGCTCAGTTCCGCCGAAGATTG
5-KpnI-RodZ1-155-U-PBAD18	GGGCTAGCGAATTCGAGCTCGGTACCATGAATACTGAAGCCACGCA
5-GFP-RodZ1-155-U-KpnI	TATACAAAGAATTCGAGCTCGGTACCAATACTGAAGCCACGCACGACC
5-MalF13-RodZ	GTCATTAAAAAGAAACATTGGTGGCAAAGCGACGCGCTGTGGCTGATGACCTTCACttg
3-MalF13-RodZ	CAGCGCGTCGCTTTGCCACCAATGTTTCTTTTTAATGACTTCCAGCCCTGGCAGCAGTT
3-XbaI-RodZ-1-155-U	TTGCATGCCTGCAGGTCGACTCTAGATTAGTTACTCAGTTCCGCCG
MalF13-UP-DOWN	GGTGGCAAAGCGACGCGCTGGATGTCATTAAAAAGAAACATTG
MalF13-DOWN-UP	CAATGTTTCTTTTTAATGACATCCAGCGCGTCGCTTTGCCACC
5-KpnI-rodZ-U	GGGCTAGCGAATTCGAGCTCGGTACCTCAATTCTCATTTAAACGTA
3-rodZ1-155-Q140K-E141K-HindIII	AAGCTTTTAGCTCAGTTCCGCCGAAGATTGATCGGCCATAGTGGTGATCTCTTtCTtCTGAGCTTTGCGGTC
5-gfp-rodZ1-155-U-KpnI	TATACAAAGAATTCGAGCTCGGTACCAATACTGAAGCCACGCACGACC
5-rodZ1-155-flag-mreC	TCTTCGGCGGAACTGAGTAACTAATCTAGAATGGATTACAAGGATGACGACGATAAG
3-rodZ1-155-flag-mreC	CTTATCGTCGTCATCCTTGTAATCCATTCTAGATTAGTTACTCAGTTCCGCCGAAGA
5-rodZ1-138-flag-mreC	GGCAAGACCGCAAAGCTTAATCTAGAATGGATTACAAGGATGAC
3-rodZ1-138-flag-mreC	GTCATCCTTGTAATCCATTCTAGATTAAGCTTTGCGGTCTTGCC
5-XhoI-t25	GACTCTCGAGCATGACCATGCAGCAATCGC
3-T25-AscI	GATTGGCGCGCCTGCAGCCCGCCGCGTGC
T18C-XbaI-mreB	gcTCTAGACTCACGAatgTTGAAAAAATTTCGTGGCAT
T18C-EcoRI-mreB	CGGAATTCTTACTCTTCGCTGAAC
5-mreC-AL-DD	TTAGAACTTGAAAACCGGGACGATCGTCAGGAACTGTTG
3-mreC-AL-DD	CAACAGTTCCTGACGATCGTCCCGGTTTTCAAGTTCTAA
5-kpnI-rodZΔHTH-gfp	CCGGTACCCTGGAAAAGCAGGCTCCACTTCGG
3-XbaI-rodZ	gcTCTAGATTACTGCGCCGGTGATT
3-mreC-305-HindIII-U	CATCCGCCAAAACAGCCAAGCTTCTAGTCTGGCGAAGGCAATA

3-mreC1-278-HindIII-U	TCTCATCCGCCAAAACAGCCAAGCTTCTAGTTAGCGCCGTTACGATC
3-HindIII-mreC-U	TCTCATCCGCCAAAACAGCCAAGCTTCTATTGCCCTCCCGGCGCAC
5-XbaI-FLAG-L-PBP2	TCTAGAATGGATTACAAGGATGACGACGATAAGGAATTCAAACTACAGAACTCTTTTCG
5-PBP2-GFP	CCGCAGCGGAGGACCATTAAATGAGTAAAGGAGAAGAACT
3-GFP-PBP2	AGTTCTTCTCCTTTACTCATTTAATGGTCCTCCGCTGCGG

References:

1. Lennox ES. 1955. Transduction of linked genetic characters of the host by bacteriophage P1. Virology 1:190-206.
2. Hamilton CM, Aldea M, Washburn BK, Babitzke P, Kushner SR. 1989. New method for generating deletions and gene replacements in Escherichia coli. J Bacteriol 171:4617-22.
3. Haldimann A, Wanner BL. 2001. Conditional-replication, integration, excision, and retrieval plasmid-host systems for gene structure-function studies of bacteria. J Bacteriol 183:6384-93.
4. Karimova G, Dautin N, Ladant D. 2005. Interaction network among Escherichia coli membrane proteins involved in cell division as revealed by bacterial two-hybrid analysis. J Bacteriol 187:2233-43.
5. Pichoff S, Vollrath B, Touriol C, Bouché JP. 1995. Deletion analysis of gene minE which encodes the topological specificity factor of cell division in Escherichia coli. Mol Microbiol 18:321-9.
6. Bernhardt TG, de Boer PA. 2003. The Escherichia coli amidase AmiC is a periplasmic septal ring component exported via the twin-arginine transport pathway. Mol Microbiol 48:1171-82.
7. Bendezu FO, de Boer PA. 2008. Conditional lethality, division defects, membrane involution, and endocytosis in mre and mrd shape mutants of Escherichia coli. J Bacteriol 190:1792-811.
8. Bendezu FO, Hale CA, Bernhardt TG, de Boer PA. 2009. RodZ (YfgA) is required for proper assembly of the MreB actin cytoskeleton and cell shape in E. coli. EMBO J 28:193-204.
9. Weiss DS, Chen JC, Ghigo JM, Boyd D, Beckwith J. 1999. Localization of FtsI (PBP3) to the septal ring requires its membrane anchor, the Z ring, FtsA, FtsQ, and FtsL. J Bacteriol 181:508-20.
10. Cho H, Wivagg CN, Kapoor M, Barry Z, Rohs PDA, Suh H, Marto JA, Garner EC, Bernhardt TG. 2016. Bacterial cell wall biogenesis is mediated by SEDS and PBP polymerase families functioning semi-autonomously. Nat Microbiol 1:16172.

11. Churchward G, Belin D, Nagamine Y. 1984. A pSC101-derived plasmid which shows no sequence homology to other commonly used cloning vectors. Gene 31:165-71.
12. Datsenko KA, Wanner BL. 2000. One-step inactivation of chromosomal genes in Escherichia coli K-12 using PCR products. Proc Natl Acad Sci U S A 97:6640-5.
13. Guzman LM, Belin D, Carson MJ, Beckwith J. 1995. Tight regulation, modulation, and high-level expression by vectors containing the arabinose PBAD promoter. J Bacteriol 177:4121-30.
